# Functional Irreplaceability of Escherichia coli and Shewanella oneidensis OxyRs Is Critically Determined by Intrinsic Differences in Oligomerization

**DOI:** 10.1128/mbio.03497-21

**Published:** 2022-01-25

**Authors:** Weining Sun, Yanlin Fan, Fen Wan, Yizhi J. Tao, Haichun Gao

**Affiliations:** a Institute of Microbiology, College of Life Sciences, Zhejiang University, Hangzhou, Zhejiang, China; b Department of BioSciences, MS-140, Rice University, Houston, Texas, USA; c College of Laboratory Medicine, Hangzhou Medical College, Hangzhou, China; California Institute of Technology; University of Michigan-Ann Arbor

**Keywords:** transcription regulation, oxidative stress response, OxyR, Gram-negative bacteria, protein structure, protein structure-function

## Abstract

LysR-type transcriptional regulators (LTTRs), which function in diverse biological processes in prokaryotes, are composed of a conserved structure with an N-terminal DNA-binding domain (DBD) and a C-terminal signal-sensing regulatory domain (RD). LTTRs that sense and respond to the same signal are often functionally exchangeable in bacterial species across wide phyla, but this phenomenon has not been demonstrated for the H_2_O_2_-sensing and -responding OxyRs. Here, we systematically examined the biochemical and structural determinants differentiating activator-only OxyRs from dual-activity ones by comparing OxyRs from two *Gammaproteobacteria*, Escherichia coli and Shewanella oneidensis. Our data show that *Ec*OxyR could function as neither an activator nor a repressor in S. oneidensis. Using *So*OxyR-based OxyR chimeras and mutants, we demonstrated that residues 283 to 289, which form the first half of the last C-terminal α-helix (α10), are critical for the proper function of *So*OxyR and cannot be replaced with the *Ec*OxyR counterpart. Crystal structural analysis reveals that α10 is important for the oligomerization of *So*OxyR, which, unlike *Ec*OxyR, forms several high-order oligomers upon DNA binding. As the mechanisms of OxyR oligomerization vary substantially among bacterial species, our findings underscore the importance of subtle structural features in determining regulatory activities of structurally similar proteins descending from a common ancestor.

## INTRODUCTION

Reactive oxygen species (ROS), including superoxide (O_2_^−^), hydrogen peroxide (H_2_O_2_), and hydroxyl radical (·OH), can damage biomolecules such as DNAs, proteins, and lipids (1). In living organisms, oxidative stress caused by ROS is inevitable because they can be generated endogenously as metabolic by-products of cellular oxygen respiration in addition to those coming from environments (1). Basal defenses in bacteria, composed of mostly ROS-scavenging enzymes, are sufficient to cope with ROS formed during routine aerobiosis. However, when intracellular levels of ROS exceed safe limits due to exogenous contribution, oxidative stress sensing and responding systems are activated to coordinately regulate expression of a set of genes to ensure that ROS concentrations are restrained at an acceptable level and damages are promptly repaired (2). The primary members of these genes encode ROS detoxification enzymes (catalases, superoxide dismutase, and various peroxidases), iron-sequestering proteins, and damage control proteins (3).

OxyR, one of the major ROS-sensing and -responding systems identified 35 years ago in enteric bacteria Escherichia coli and Salmonella enterica serovar Typhimurium (S. Typhi), is an LysR-type transcriptional regulator (LTTR) that is characterized by having an N-terminal DNA-binding domain (DBD) and a C-terminal regulatory domain (RD) (2, 4, 5). As best illustrated in E. coli, OxyR (*Ec*OxyR) becomes activated when a disulfide bond is formed between two conserved cysteine residues located in the RD as a result of oxidization by H_2_O_2_ (6–7). The structural changes in the RD induced by the disulfide bond formation lead to conformational rearrangement of the DBD with an altered DNA-binding affinity (8–9). Because *Ec*OxyR functions as an activator of its regulon only, we refer to it as a type I OxyR in this study (8).

Although it is a type I OxyR that was initially identified and studied, further investigations into its homologs from other bacteria revealed surprising variations in their functional modes. OxyRs of corynebacteria function as a repressor only (type III) for more than 20 genes, including those for ROS detoxification enzymes and iron-sequestering proteins (10, 11). Most OxyRs belong to the dual-control (type II) group, acting as not only an activator of peroxide‐scavenging enzymes under oxidative stress conditions but also a repressor of the same target genes under nonstress conditions. Type II OxyRs occur in a large variety of bacteria, such as *Shewanella*, Pseudomonas, *Neisseria*, *Xanthomonas*, and *Deinococcus*, to name a few (12–17).

Intriguingly, despite the differences in functional modes, OxyRs characterized to date are similar in overall structure and recognize similar DNA motifs composed of two tandem ATAG-N_7_-CTAT (N represents any nucleotide) repeats with a 7- to 10-bp interval (4, 9, 15, 18, 19). Moreover, all OxyRs examined to date, regardless of their regulatory effects, are capable of binding to promoter regions of their target genes in both reduced and oxidized forms (9, 15, 19, 20). It has been proposed that as a repressor (in the reduced state), OxyRs bind to a more extended region in proximity of the core DNA motifs than in the oxidized state, thus occluding RNA polymerase binding (21). However, given that OxyRs in reduced and oxidized forms coexist in the cell, recent reports have provided evidence to suggest that OxyRs in both redox states interact with the same DNA sequence but differ from each other in binding affinity (15, 19).

Despite overall similarities in sequences, structures, and activation mechanisms, bacterial OxyRs are generally not interchangeable, with exceptions of the same type from the same or closely related species (5, 15). While it has been suggested that the functional irreplaceability is a result of intrinsic structural differences among OxyR orthologues (15, 22), up until now, little is known about the underlying mechanisms.

In this study, we attempted to unravel mechanisms for functional irreplaceability by carrying out comparative analyses of type I *Ec*OxyR and type II OxyR of Shewanella oneidensis, a representative of a large group of Gram-negative facultative *Gammaproteobacteria* renowned for their respiratory versatility and the potential application in biogeochemical circulation of minerals and bioelectricity (23, 24). We demonstrate that *Ec*OxyR could function neither as an activator nor a repressor in S. oneidensis. The crystal structure of the *So*OxyR RD in its reduced form shows an antiparallel dimer similar to OxyRs from E. coli and other bacterial species. It is also observed that *So*OxyR RD dimers further interact through an α-helix at the C terminus to form tetramers and other high-order oligomers. Indeed, *So*OxyR/*Ec*OxyR chimeras and *So*OxyR mutants demonstrated that the last α-helix of *So*OxyR is essential for its proper regulatory activity. Furthermore, DNA gel shift assays indicated that *So*OxyR has a much stronger tendency than *Ec*OxyR to form oligomeric assemblies, which is presumably due to cooperative DNA binding mediated by the last α-helix as revealed in the crystal structure of *So*OxyR. Overall, our findings provide a mechanistic explanation for the functional nonexchangeability between *So*OxyR and *Ec*OxyR, and they highlight the importance of OxyR oligomerization, the mode of which may vary widely among related bacterial species, on the regulatory activity of these transcriptional regulators.

## RESULTS

### *Ec*OxyR has no physiological activity in S. oneidensis.

We have previously shown that both S. oneidensis and E. coli *oxyR* null mutants exhibit severe plating defects on LB plates (substantially impaired viability) (17, 25) (Fig. S1A in the supplemental material). Functional nonexchangeability between *So*OxyR and *Ec*OxyR was demonstrated, as they failed to reciprocally complement the phenotypes of the opposite *oxyR* mutant (17). Unlike type II *So*OxyR, which functions as both repressor and activator for some of its regulon members such as *katB*, type I *Ec*OxyR could not activate expression of these genes (17, 19). Given that the repressing activity of OxyRs could not be detected from cell viability when growing on agar plates, we set out to determine whether *Ec*OxyR could repress expression of the *katB* gene.

10.1128/mbio.03497-21.1 FIG S1 The DBD of OxyRs alone is not functional. (A) Droplet assays for viability and growth assessment. Cultures of indicated strains prepared to contain approximately 10^9^ CFU/mL were regarded as the undiluted (dilution factor, 0), which were subjected to 10-fold series dilution. Five microliters of each dilution was dropped on LB plates. Results were recorded after 24 h incubation. pOxyR represents that each strain expresses its own *oxyR* gene. (B) Validation of expression of *EcOxyR* and *SoOxyR* with GFP fusions. DNA constructs were placed under control of the *SoOxyR* promoter and integrated into the chromosome to allow expression from a single copy. Cells of the mid-exponential phase were visualized. (C) GFP quantification analysis of GFP fused to indicated OxyR variants. Error bars show standard deviations. In panels A and B, experiments were performed at least three times, with representative results being presented. Download FIG S1, PDF file, 0.2 MB. Copyright © 2022 Sun et al. 2022 Sun et al. https://creativecommons.org/licenses/by/4.0/ This content is distributed under the terms of the Creative Commons Attribution 4.0 International license.

**FIG 1:**
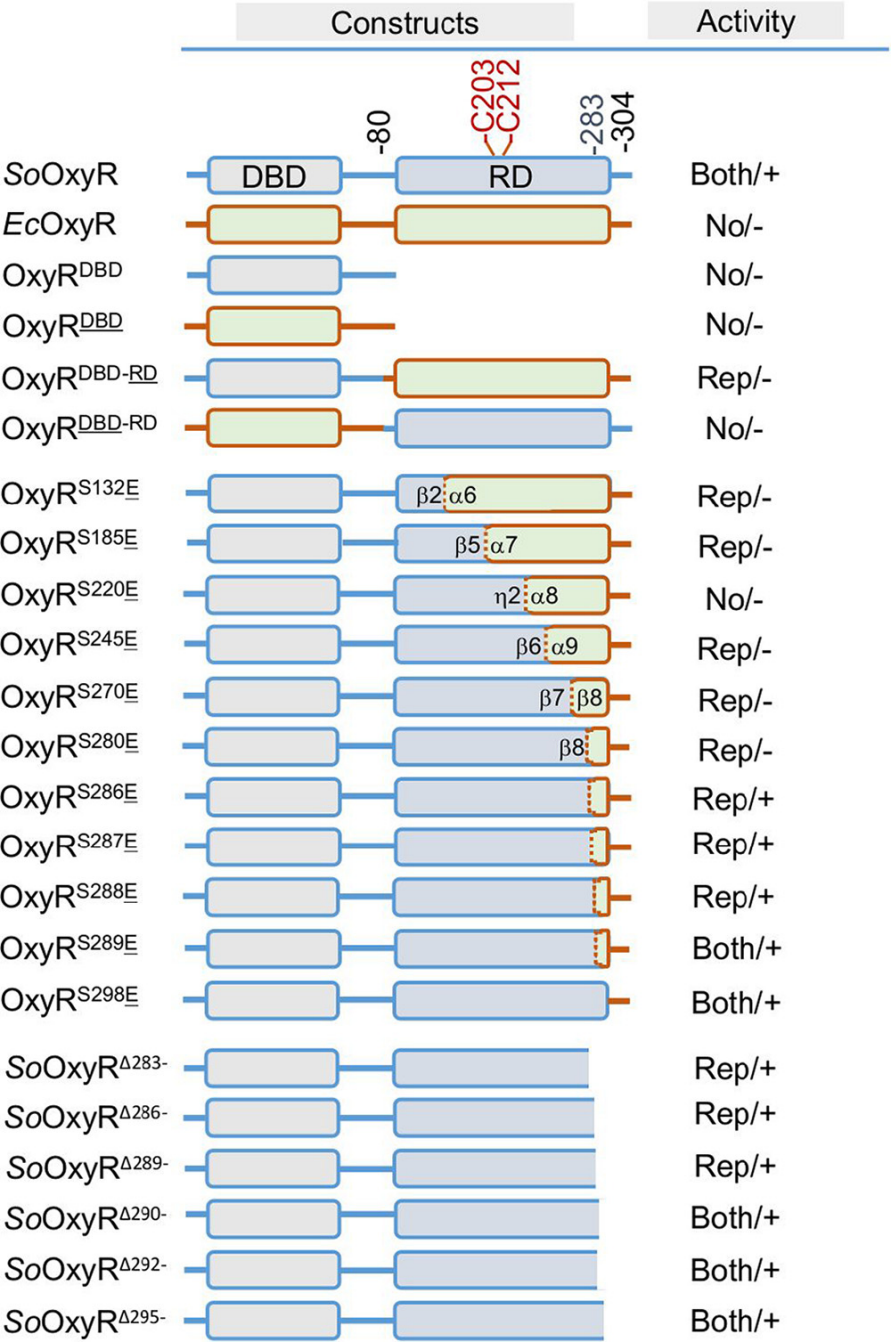
OxyR variants of gene fusion and truncations. DNA constructs were placed under the control of the *SoOxyR* promoter and integrated into the chromosome to allow expression from a single copy. Residues that are redox active and act as a location marker were shown. There were two parameters for activity, physiological impacts and response to H_2_O_2_. The former is represented by “Both” (repressing and activating), “No” (no effect), “Rep” (repressing), and “Act” (activating), while the latter is represented by positive (+) and negative (−) symbols. Point mutation variants are given in Table S2 in the supplemental material.

**FIG 2:**
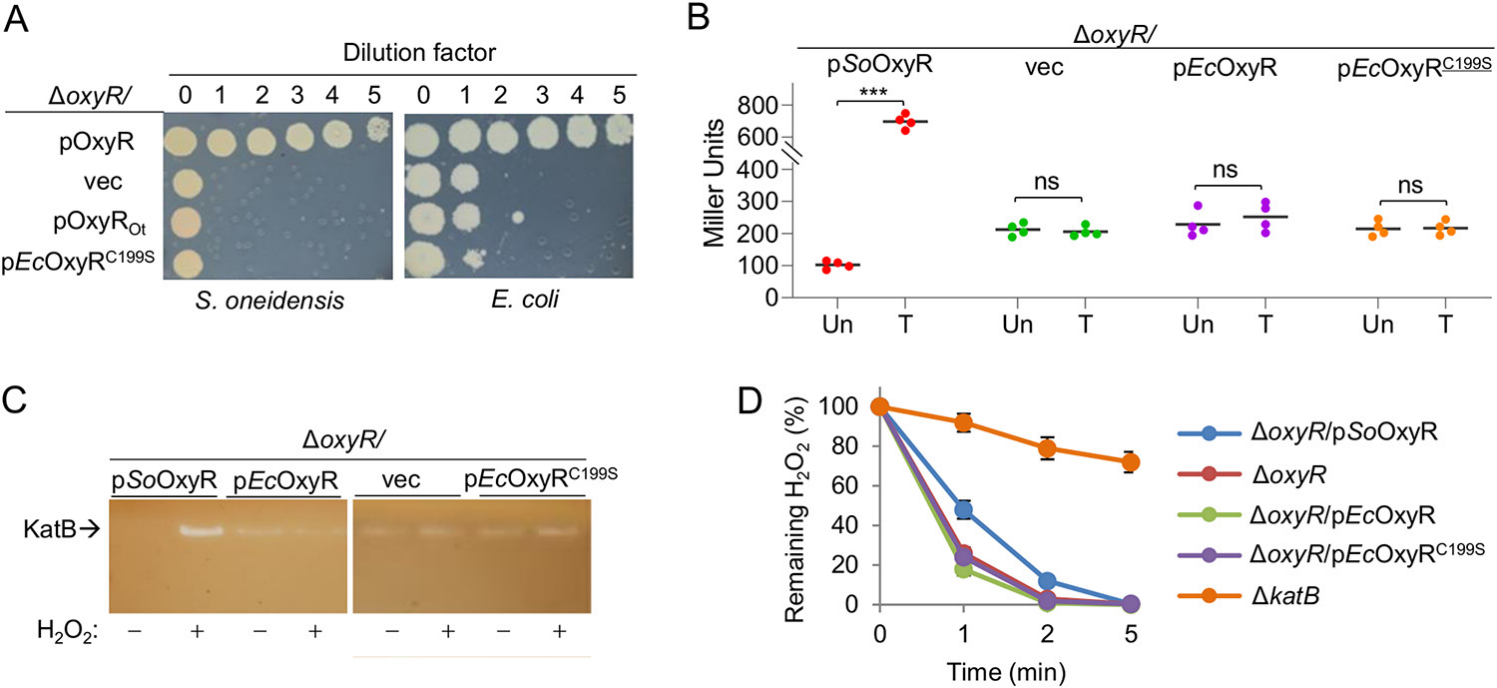
*Ec*OxyR has no physiological activity in S. oneidensis. (A) Droplet assays for viability and growth assessment. Cultures of indicated strains prepared to contain approximately 10^9^ CFU/mL were regarded as the undiluted (dilution factor, 0), which were subjected to 10-fold serial dilution. Five microliters of each dilution was dropped onto LB plates. Results were recorded after 24 h of incubation. Expression of the *oxyR* genes was driven by the *SoOxyR* promoter. pOxyR and pOxyR_Ot_ represents that each strain expresses *oxyR* of its own or of the other’s. (B) Impacts of OxyRs on expression of *katB* by using integrative *lacZ* reporters. Cells at the mid-exponential phase were used for all assays unless otherwise noted. Cells directly taken as untreated (Un) and incubated with 0.2 mM H_2_O_2_ for 2 min as treated (T). Asterisks indicate statistically significant difference of the values compared (*n *= 4, *, *P < *0.05; **, *P < *0.01; ***, *P < *0.001). (C) Catalase detected by staining and activity assay. Cells were either directly used or incubated with 0.2 mM H_2_O_2_ for 30 min. Cell lysates containing the same amount of protein were subjected to 10% nondenaturing PAGE. Catalase (KatB) was revealed by catalase staining as described in Materials and Methods. (D) For H_2_O_2_ degradation assay, cells were adjusted to the same optical density and disrupted by sonication. The resultant cell extracts were mixed with 1 mM H_2_O_2_ and assayed for the remaining H_2_O_2_ in the reaction mixture at the indicated time points, which was normalized to give relative amounts to the original (100%). Experiments were performed at least three times or specified as in panel B, with representative results (A and C) or the average ± error bars representing standard deviation being presented (D).

DNA fragments for both *Ec*OxyR and *So*OxyR, as well as all OxyR variants used in this study, were amplified and cloned into integrative vector pHGM01 (26). Protein constructs for OxyR variants tested in this study, including truncations, chimeric hybrids, and point mutants, are shown in Fig. 1, with additional information given in Table S1. Throughout the study, point mutations were presented in subscript, and all others were presented in superscript. The verified vectors were then introduced into the Δ*SooxyR* strain for chromosomal integration, resulting in strains with the *oxyR* variants under the control of the *oxyR* promoter (P*_oxyR_*). In this way, all OxyR variants under test are supposed to be produced at levels similar to that of OxyR in the wild type (WT). Indeed, expression of *Ec*OxyR and *So*OxyR was found to be comparable by using fusion proteins with green fluorescent protein (GFP) linked to the C terminus, which could be detected in the cytoplasm by confocal microscopy (Fig. S1B and C). Because WT and the Δ*oxyR* strains of S. oneidensis and E. coli expressing a copy of their own *oxyR* gene integrated into the chromosome, namely, Δ*SooxyR*/p*So*OxyR and Δ*EcoxyR*/p*Ec*OxyR, respectively, were indistinguishable from each other in all experiments (Fig. S1A), only data for the Δ*SooxyR*/p*So*OxyR strain (regarded as WT) are presented (Fig. 2A).

10.1128/mbio.03497-21.8 TABLE S1 Strains, plasmids, and all OxyR variants used in this study. Download Table S1, PDF file, 0.2 MB. Copyright © 2022 Sun et al. 2022 Sun et al. https://creativecommons.org/licenses/by/4.0/ This content is distributed under the terms of the Creative Commons Attribution 4.0 International license.

10.1128/mbio.03497-21.9 TABLE S2 Crystallographic data collection and refinement statistics. Download Table S2, PDF file, 0.1 MB. Copyright © 2022 Sun et al. 2022 Sun et al. https://creativecommons.org/licenses/by/4.0/ This content is distributed under the terms of the Creative Commons Attribution 4.0 International license.

We then compared *katB* expression levels in Δ*SooxyR* cells expressing either *So*OxyR or *Ec*OxyR with integrative *lacZ* reporters used before (19). As expected, Δ*SooxyR*/p*So*OxyR exhibited a repressing effect on *katB* expression in normal growing cells up to the mid-exponential phase and substantially elevated expression levels after cells were challenged by H_2_O_2_ (Fig. 2B). In the absence of *So*OxyR, the *katB* gene was expressed at levels between the repressed and the activated caused by the regulator. Clearly, Δ*SooxyR*/p*Ec*OxyR neither exhibited an H_2_O_2_-responsive effect nor showed any repressing activity in S. oneidensis (Fig. 2B). This was not due to the shortage of the reduced proteins because *Ec*OxyR_C199S_, a sensory residue point mutant locked in the reduced form, exhibited the same effect (Fig. 2B). These observations were supported by catalase staining analysis of cells prepared similarly, as KatB is the only catalase detectable by the method (17). Neither *Ec*OxyR nor *Ec*OxyR_C199S_ could affect KatB levels in Δ*SooxyR* cells (Fig. 2C). Furthermore, the effects of *Ec*OxyR and *Ec*OxyR_C199S_ on H_2_O_2_ degradation of the Δ*SooxyR* strain were assessed. Cells expressing proteins of interest at the mid-exponential phase were collected and disrupted by sonication in order to avoid interference of H_2_O_2_ induction on catalase expression and of peroxidases which require electron transport. The resultant cell extracts were aliquoted, adjusted to contain the same amount of protein, and mixed with 1 mM H_2_O_2_ for assaying the remaining H_2_O_2_ in the reaction at the indicated time points. In line with the failure of suppressing the plating defect, neither *Ec*OxyR nor *Ec*OxyR_C199S_ had a significant impact on H_2_O_2_ degradation (Fig. 2D). All together, these data indicate that *Ec*OxyR does not have detectable repressing and activating activity in S. oneidensis.

### DBD of *So*OxyR is essential for repressing activity in S. oneidensis.

Given that the regulatory activity of OxyR is ultimately realized by interaction between the DBD domain and its target DNAs, we set out to determine whether the DBD domain could function to some extent on its own. For simplicity, *So*OxyR and *Ec*OxyR fragments were presented in regular and underlined superscript, respectively; for example, OxyR^DBD^ (truncated mutation) and OxyR^DBD^ represent the DBD domain of *So*OxyR and *Ec*OxyR, respectively (Fig. 1). Characterization of OxyR^DBD^ and OxyR^DBD^ demonstrated that the DBD domain of OxyRs alone does not possess any regulatory activity in S. oneidensis (Fig. S2A and B).

10.1128/mbio.03497-21.2 FIG S2 The DBD domain of OxyRs. The DBD domain of OxyRs has no activity in S. oneidensis, supported as follows. (A) Droplet assays for viability and growth assessment. OxyR^DBD^ and OxyR^DBD^ represent the DBD domains of *So*OxyR and *Ec*OxyR, respectively (refer to the text for details). (B) Impacts of the DBD domains of OxyRs on expression of *katB* by using integrative *lacZ* reporters. Cells at the mid-exponential phase were used for all assays unless otherwise noted. Cells directly taken as untreated (Un) and incubated with 0.2 mM H_2_O_2_ for 2 min and treated (T). DBD of *So*OxyR is essential for repressing activity in S. oneidensis, supported as follows. (C) Droplet assays for viability and growth assessment. (D) H_2_O_2_ degradation assay. Experiments were performed at least three times, with the average ± error bars representing standard deviation or representative results being presented. Download FIG S2, PDF file, 0.2 MB. Copyright © 2022 Sun et al. 2022 Sun et al. https://creativecommons.org/licenses/by/4.0/ This content is distributed under the terms of the Creative Commons Attribution 4.0 International license.

**FIG 3:**
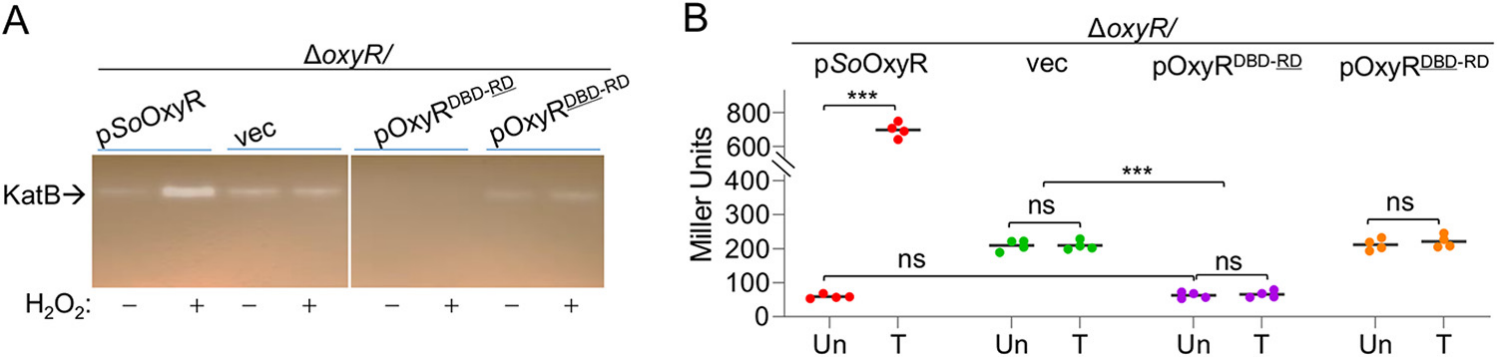
DBD of *So*OxyRs is essential for repressing activity in S. oneidensis. (A) Catalase detected by staining and activity assay. Experiments were performed at least three times, with representative results being presented. (B) Impacts of OxyRs on expression of *katB* by using integrative *lacZ* reporters. Asterisks indicate statistically significant difference of the values compared (*n *= 4; *, *P < *0.05; **, *P < *0.01; ***, *P < *0.001).

To investigate the mechanism for functional differences between *So*OxyR and *Ec*OxyR, we constructed hybrid protein OxyR^DBD-^^RD^ (*So*OxyR DBD and *Ec*OxyR RD) (Fig. 1). Clearly, OxyR^DBD-^^RD^ did not elicit significant difference in viability (Fig. S2C), indicating that it could not function as an activator for the *So*OxyR regulon. Indeed, catalase staining revealed that catalase production was not induced in cells expressing this hybrid OxyR when challenged by H_2_O_2_ (Fig. 3A). Moreover, the KatB levels in Δ*SooxyR* cells producing OxyR^DBD-^^RD^ were indistinguishable from that of WT, suggesting that OxyR^DBD-^^RD^ could function as a repressor (Fig. 3A). This notion was supported by results from the expression assay (Fig. 3B): OxyR^DBD-^^RD^ repressed *katB* expression and was not responsive to H_2_O_2_ treatment, and results from H_2_O_2_ degradation assay (Fig. S2D) revealed reduced H_2_O_2_ removal rates for the Δ*SooxyR* strains producing OxyR^DBD-^^RD^. Thus, OxyR^DBD-^^RD^ can function as a repressor but not an activator for expression of the *So*OxyR regulon. Hybrid protein OxyR^DBD^^-RD^ (E. coli DBD and S. oneidensis RD) was then constructed for cross-examination (Fig. 1). OxyR^DBD^^-RD^ was not responsive to H_2_O_2_ as expected (Fig. 3A) and could not correct the plating defect of either Δ*SooxyR* or Δ*EcoxyR* strains (Fig. S2C). Moreover, this protein differed from OxyR^DBD-^^RD^ in that it did not repress KatB production (Fig. 3A and B; Fig. S2D). These data altogether indicate that the *So*OxyR DBD domain is essential for the repressing effect of *So*OxyR, implying that the mechanisms underlying the functional difference between type I and type II OxyRs are more profound than expected.

### The crystal structure of *So*OxyR^C203S^ RD reveals a reduced redox center and potential to oligomerize.

**FIG 4:**
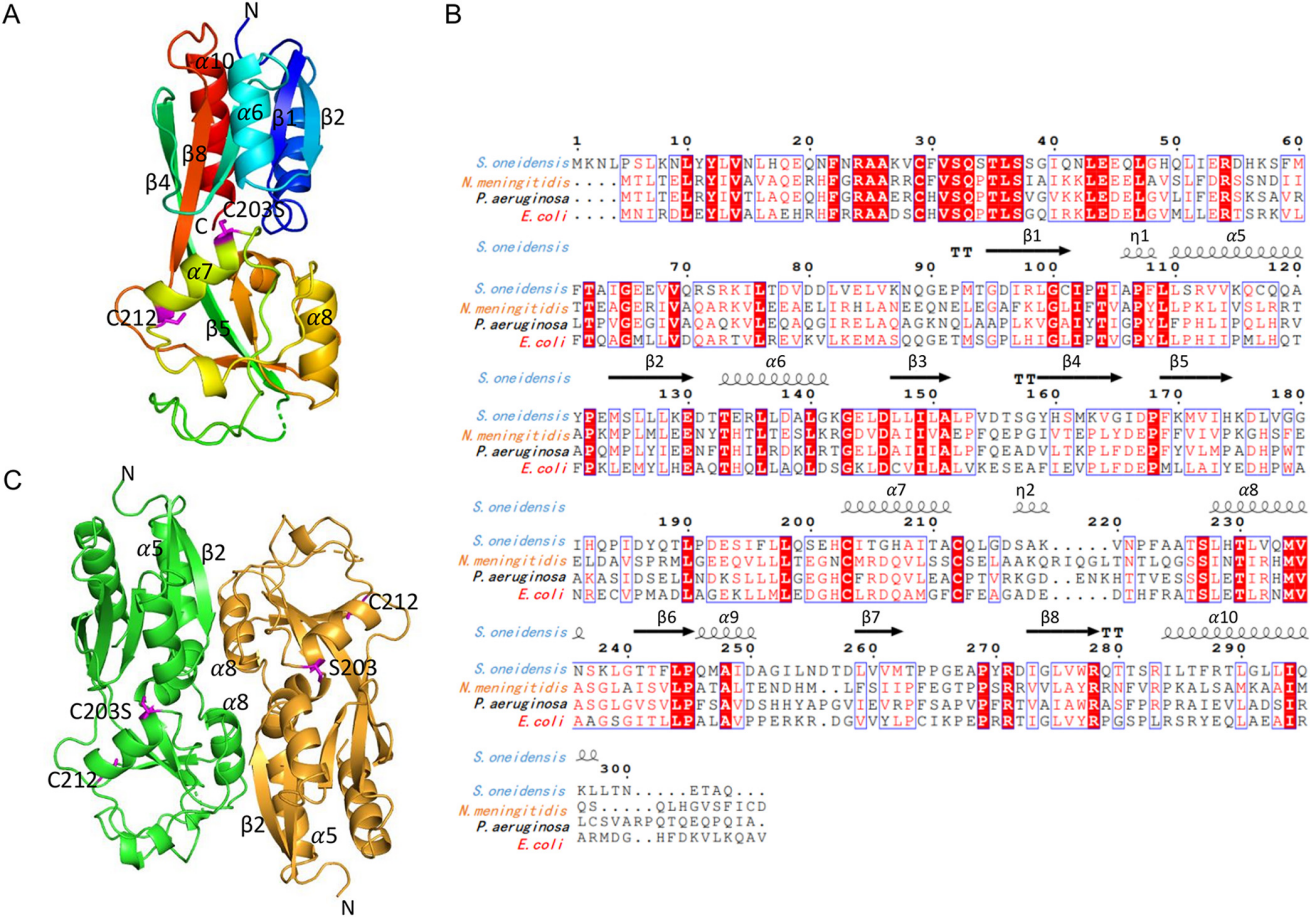
Crystal structure of *So*OxyR RD_C203S_. (A) A monomer. The molecule is rainbow colored with the N terminus in blue and C terminus in red. (B) Multisequence alignment and secondary structure assignment. β-Strands and α-helices are shown by arrows and coils, respectively. Secondary structures are numbered assuming that the DBD domain of *So*OxyR would have a similar structure as *Pa*OxyR (PDB ID 2X6G). In panel A, the secondary structure elements, along with the N/C termini and the redox residue pair, are labeled. The redox residue pair C203S and C212 are highlighted in magenta using sticks representation. (C) Dimer structure. The two subunits are shown in green and orange, respectively.

To better understand why *Ec*OxyR cannot function as a replacement of *So*OxyR, we determined the crystal structure of the RD_C203S_ of *So*OxyR, which should be locked in the reduced form with a single point mutation C203S (17), as the mutant could no longer form the disulfide bond C203-C212. The crystal structure of RD_C203S_ of *So*OxyR was solved to a resolution of 2.4 Å by molecular replacement (Fig. 4A; Table S2). The RD_C203S_ of *So*OxyR polypeptide chain, which contains residues 91 to 304 of the full-length protein, was traced unambiguously except for the last four residues at the C terminus and a six-residue loop from amino acids (aa) 179 to aa184 that were structurally disordered. Each RD_C203S_ molecule is made of six α-helices and eight β-strands that fold into two domains, RD-I (residues 91 to 164 and 274 to 304) and RD-II (residues 165 to 273). RD-I is made of a central five-stranded β-sheet with two α-helices stacked against one face and one α-helix against the other. RD-II, which hosts the redox center, is comprised of a central three-stranded β-sheet that is surrounded by three loosely organized α-helices. RD-I and RD-II are connected by two loop linkers comprised of residues 165 to 168 and 264 to 273, with an extensive interdomain interface that is primarily mediated by two helices (i.e., α7 and α9) from RD-II and several loops from RD-I (Fig. 4A and B). Multisequence alignment indicates that RD-I is better conserved within the LysR family in primary sequences compared to RD-II (Fig. 4B). It is also evident that RD-II has a much higher fraction of structured loops (Fig. 4B). According to a DALI search, the structure of the reduced S. oneidensis OxyR RD monomer is closely related to those of the reduced OxyR2 of Vibrio vulnificus (PDB ID 5X0V; Z score, 31.5; root mean square deviation [RMSD], 1.2 Å), the Pseudomonas aeruginosa full-length OxyR _C199D_ (PDB ID 4X6G; Z score, 23.5; RMSD, 2.2 Å), the reduced P. aeruginosa OxyR RD (PDB ID 4Y0M, Z score, 23.5; RMSD, 2.4 Å), the RD_C199S_ of *Ec*OxyR (PDB ID 1I69, Z score, 22.3; RMSD, 2.3 Å), and the RD of reduced Neisseria meningitides OxyR (PDB ID 3JV9; Z score, 21.6; RMSD, 2.3 Å), with no major differences in conformation.

As expected for a reduced form, the disulfide residue pair C203S and C212 in *So*OxyR RD are found at the opposite ends of the helix α7, with the −OH and the −SH side chain measured to be 13 Å apart from each other. The side chains of both C203S and C212 point away from solvent by tucking into cavities that are partially polar for C203S but completely nonpolar for C212. Based on the structural context, the thiol side chain of residue 203 in a wild-type protein is likely to be more accessible for oxidative modification than C212. In the reduced *Ec*OxyR RD, the side chain of C199S is also tucked in as in *So*OxyR RD, but the side chain of C208 is exposed to solvent due to a sharp bend in the middle of the helix connecting the redox pair residues (27) (Fig. S3A to C). It is worth noting that the positioning of the two redox pair residues in the *Vv*OxyR2 RD, also in the reduced form, is almost identical to *So*OxyR (27). Like *Vv*OxyR2, *So*OxyR has an -EH- dipeptide in front of C203 instead of a -GH- found in *Ec*OxyR. A previous study on *Vv*OxyR2 indicated that the glutamic acid residue is able to enhance H_2_O_2_ sensitivity (27). The active site residues surrounding C203S, including T104, T133, H202, and R271, are also conserved in *So*OxyR as in *Vv*OxyR2 (11).

10.1128/mbio.03497-21.3 FIG S3 Structural comparison of the S. oneidensis OxyR and E. coli OxyR RD. (A) The reduced forms of S. oneidensis OxyR^C203S^ RD (light green) and E. coli OxyR^C199S^ RD (PDB ID 1I69; light pink) superimposed. The redox-active cysteine residues C203S/C212 in *So*OxyR^C203S^ RD are shown in the space-filling model and colored in green. The redox-active cysteine residues C199S/C208 in *Ec*OxyR^C199S^ RD are also shown in the space-filling model but colored in red. (B) Pairwise structure comparison of *So*OxyR^C203S^ RD (light green) and the oxidized form of *Ec*OxyR RD (PDB 1I6A; yellow). The disulfide-linked C199-C208 in oxidized *Ec*OxyR RD is highlighted in orange. (C) Comparison of the redox active centers of “reduced” *So*OxyR^C203S^ (light green), reduced form of *Ec*OxyR^C199S^ (light pink), and oxidized form of E. coli OxyR (yellow). Redox-active cysteine residues C203S/C212 and C199/C208 in *So*OxyR^C203S^ and *Ec*OxyR RD, respectively, are shown in sticks representation and colored the same as in panels A and B. Download FIG S3, PDF file, 0.5 MB. Copyright © 2022 Sun et al. 2022 Sun et al. https://creativecommons.org/licenses/by/4.0/ This content is distributed under the terms of the Creative Commons Attribution 4.0 International license.

**FIG 5:**
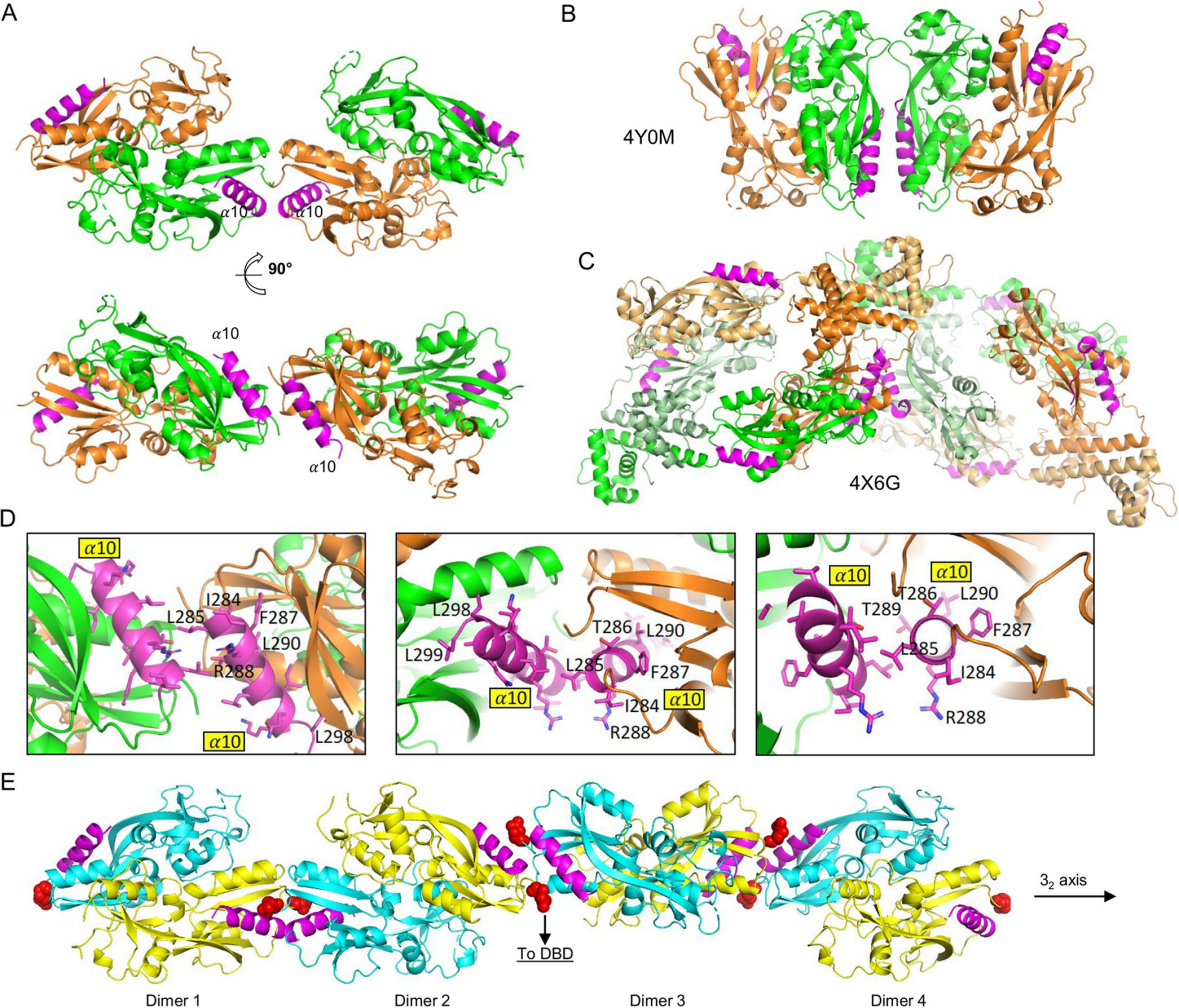
*So*OxyR RD_C203S_ tetramer. (A) Side view and top view of *So*OxyR RD_C203S_ tetramer. The tetramer is made of a dimer of dimers. The two subunits within each dimer are colored differently in green and orange. The α10 helix at the tetramer interface is highlighted in magenta. (B) Tetramer found in the crystal of reduced *Pa*OxyR RD (PDB ID 4Y0M). (C) Tetramer found in the crystal of the full-length *Pa*OxyR (PDB ID 4X6G). (D) Magnified view of the *So*OxyR RD_C203S_ tetramer interface mediated by α10. The side chains of residues from α10 are shown in sticks representation and labeled. The left panel is a top view, whereas the middle and right panels are two slightly different side views. (E) The continued polymerization of *So*OxyR dimers through the RD domain could lead to the formation of tetramers, hexamers, octomers, and so on. These RD dimers are related by a crystallographic 3_2_ symmetr*y* axis as indicated. α10, which plays a critical role in mediating polymerization, is highlighted in magenta. Residue 91, which is directly connected to the DBD domain, is shown as red spheres. Therefore, dimerization by α10 helps to bring two DBD domains into proximity to facilitate DNA binding.

There are six *So*OxyR RD_C203S_ molecules in each crystallographic asymmetric unit (Fig. S4A). Using the PDBePISA server (https://www.ebi.ac.uk/msd-srv/prot_int/cgi-bin/piserver), which considers both noncrystallographic and crystallographic interactions, a series of intermolecular interfaces are identified (Table S3). Through interface-I, RD_C203S_ monomers assemble into dimers with an average buried surface area of 1,268 Å^2^. This dimer interface, which is primarily mediated by α5, loop aa219-223 and α8 (Fig. 4C; Fig. S4B), is commonly observed in other bacterial OxyR RD crystal structures (9, 18). The second-largest interface (i.e., interface II), which buried a surface area of 561 Å^2^ on average, further assembles RD_C203S_ dimers into tetramers or dimers of dimers. Interface II is mediated by α10 located at the very C-terminal end of the protein (Fig. 5A; Fig. S4C). α10 from two neighboring subunits pack against each other in an antiparallel manner, forming an interface that is largely hydrophobic in nature (Fig. 5A and D). The *So*OxyR RD_C203S_ tetramer arrangement differs considerably from the tetrameric structure of the full-length *Pa*OxyR (PDB ID 4X6G) in which the second dimer interface is mediated by the DBD domain. Nevertheless, the C-terminal α-helix of *Pa*OxyR is implicated in high-order molecular interactions in both full-length C199D and the reduced *Pa*OxyR RD crystal structures (Fig. 5B and C), suggesting that the C-terminal α-helix may also play a biological role in OxyR oligomerization and gene regulation. The C-terminal α-helix in the two *Pa*OxyR structures makes parallel interaction, whereas this C-terminal α-helix makes antiparallel interactions in the *So*OxyR RD_C203S_ structure. It is worth noting that of the six RD_C203S_ molecules in each crystal asymmetric unit (Fig. S4A), molecular pairs A and C, B and D, and E and F can each assemble into an infinitely long helical fiber through alternating interface I and interface II dimer interactions around the 3_2_ crystallographic symmetry (Fig. 5E).

10.1128/mbio.03497-21.4 FIG S4 Analysis of intermolecular interfaces. (A) Six molecules in one crystallographic asymmetric unit. The six molecules are colored differently and labeled from A to E. (B) Dimer by interface I. (C) Dimer by interface II. (D) Dimer by interface III. The color scheme is consistent from panels A to D. Download FIG S4, PDF file, 0.4 MB. Copyright © 2022 Sun et al. 2022 Sun et al. https://creativecommons.org/licenses/by/4.0/ This content is distributed under the terms of the Creative Commons Attribution 4.0 International license.

10.1128/mbio.03497-21.10 TABLE S3 Molecular interface list calculated by PDBePISA. Download Table S3, PDF file, 0.2 MB. Copyright © 2022 Sun et al. 2022 Sun et al. https://creativecommons.org/licenses/by/4.0/ This content is distributed under the terms of the Creative Commons Attribution 4.0 International license.

In addition to the two interfaces mentioned above, interface III, ∼479 Å^2^ in size, creates another dimer through interactions mediated primarily by the redox helix (i.e., helix α7) (Fig. S4D). Because interface III is smaller than the first two, and also because this interaction is only observed between two out of the six molecules in a crystal asymmetric unit, we consider it not stable and, therefore, not biologically important. Other interfaces identified by PDBePISA are increasingly weaker and also asymmetric in nature and, therefore, were not considered further in this study.

### Fragmentation effect of the RD domains of *So*OxyR and *Ec*OxyR for functional exchangeability.

**FIG 6:**
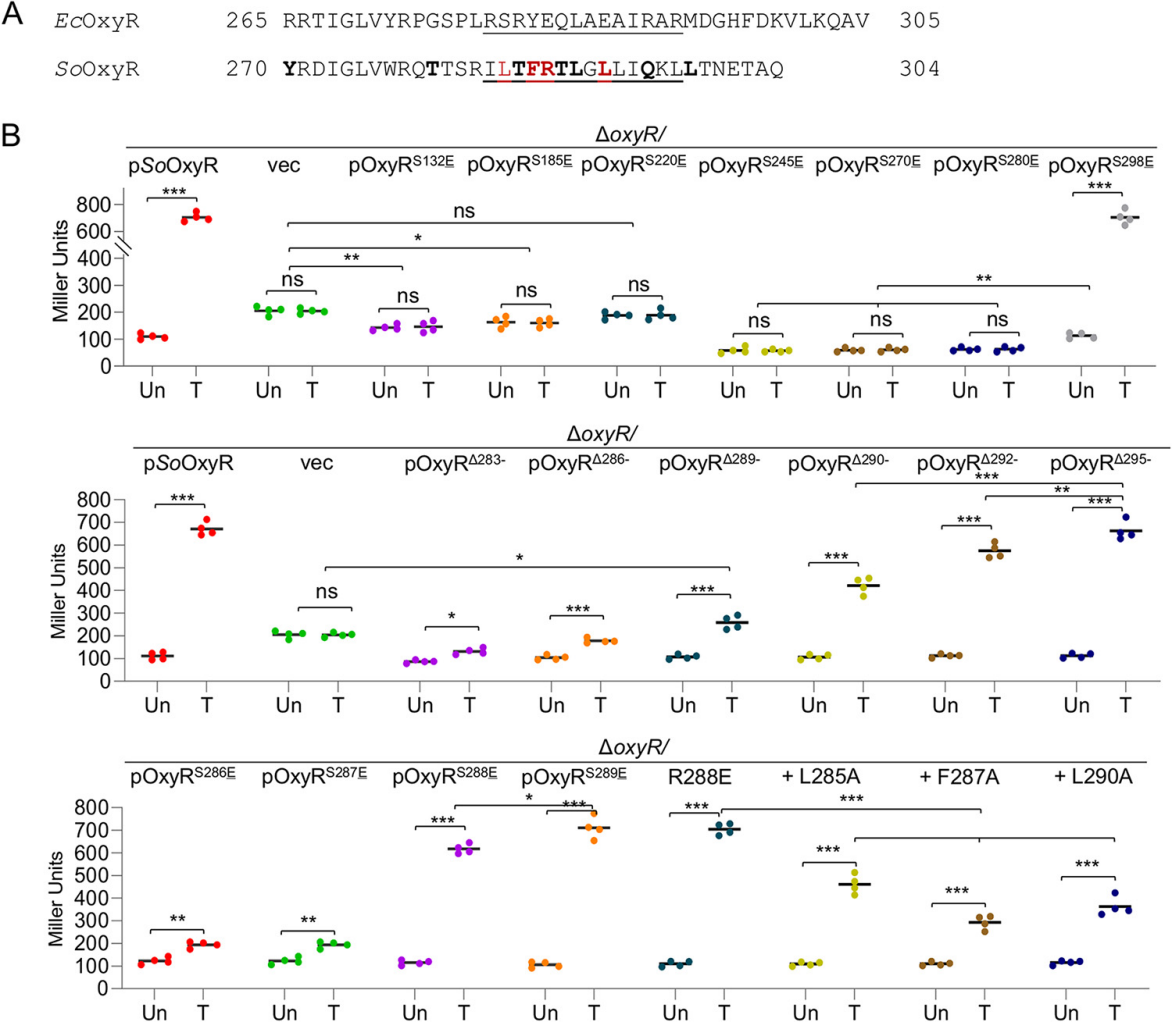
Impacts of residues within the last α-helix of *So*OxyR. (A) Local sequence alignment for the last α-helix (α10, underlined) region from *So*oxyR and *Ec*OxyR. Residues in bold (mutated to the E. coli counterparts) and/or in color (alanine scanning) were subjected to point mutation analysis. (B) Impacts of OxyR mutants on expression of *katB* by using integrative *lacZ* reporters. Cells at the mid-exponential phase were collected for the assay. Asterisks indicate statistically significant difference of the values compared (*n *= 4; *, *P < *0.05; **, *P < *0.01; ***, *P < *0.001; ns, not significant). The difference between P*So*OxyR and pOxyR^Δ295−^ was not significant.

Given the overall structural similarity in the RD domain of OxyRs, we attempted to determine the maximal length of the *So*OxyR RD domain that could be replaced by its E. coli counterpart without affecting its regulatory activity. Based on the structure comparison, the following 7 hybrid proteins were constructed without disrupting secondary structure elements: OxyR^S132^^E^ (chimera protein, *So*OxyR and *Ec*OxyR sequences before and after residue 131, respectively; between β2 and α6), OxyR^S185^^E^ (between β5 and α7), OxyR^S220^^E^ (between η2 and α8), OxyR^S245^^E^ (between β6 and α9), OxyR^S270^^E^ (between β7 and β8), OxyR^S280^^E^ (between β8 and α10), and OxyR^S298^^E^ (after α10) (Fig. 1; Table S1). Among these chimeric OxyRs, only OxyR^S298^^E^ displayed full activity of *So*OxyR (Fig. 6A and B; Fig. S5A), suggesting that the vast majority of the RD domain contributes to functional nonexchangeability. The ability of OxyR^S298^^E^ to respond to H_2_O_2_ was confirmed by catalase staining (Fig. S5B), implying that the fragment between residues 280 and 298 is crucial for the activating effect of *So*OxyR. Although all other chimeras were either nonresponsive to H_2_O_2_ or unable to function as an activator in S. oneidensis, they could be divided into two groups. The first three, OxyR^S132^^E^, OxyR^S185^^E^, and OxyR^S220^^E^, showed some repressing activity, albeit not as robust as *So*OxyR. Clearly, the shorter the E. coli fragments in the chimeras, the less the repressing effect. Despite this, their impacts on viability of Δ*SooxyR* cells were not apparent (Fig. S5A). The next three, OxyR^S245^^E^, OxyR^S270^^E^, and OxyR^S280^^E^, exhibited repression even stronger than *So*OxyR, similar to OxyR^DBD-^^RD^ (Fig. 6B). Consistently, OxyR^S245^^E^, OxyR^S270^^E^, and OxyR^S280^^E^ further sensitized cells of Δ*SooxyR* on the LB agar plates (Fig. S5A), clearly due to lowered catalase production (Fig. S5B). This observation suggests that the fragment of *Ec*OxyR RD after residue 245 introduces an impact on repressing activity of OxyR stronger than its *So*OxyR counterpart. Thus, the RD domains of *So*OxyR and *Ec*OxyR appear to affect activity in a manner of fragmentation: the RD domain is composed of multiple fragments, and each of them primarily associates with a specific activity, altogether amounting to the terminal effect of OxyR.

10.1128/mbio.03497-21.5 FIG S5 Fragmentation effect of the RD domains of *So*OxyR and *Ec*OxyR for functional exchangeability. (A) Droplet assays for viability and growth assessment. (B) Catalase detected by staining and activity assay. Cells were either directly used or incubated with 0.2 mM H_2_O_2_ for 30 min. Experiments were performed at least three times, with representative results being presented. Download FIG S5, PDF file, 0.2 MB. Copyright © 2022 Sun et al. 2022 Sun et al. https://creativecommons.org/licenses/by/4.0/ This content is distributed under the terms of the Creative Commons Attribution 4.0 International license.

### Impacts of residues within the last α-helix of *So*OxyR.

The substantial difference in activity between OxyR^S280^^E^ and OxyR^S298^^E^ prompted us to focus on α10, which is implicated in tetramer assembly based on the crystal structure of the *So*OxyR RD. A serial of truncations of *So*OxyR was generated, from *So*OxyR^Δ283−^ (truncation lacking all residues after R283) to *So*OxyR^Δ295−^, with each increasingly longer by 3 residues (Fig. 1; Table S1). Characterization of these truncations revealed that *So*OxyR^Δ295−^ and *So*OxyR^Δ292−^ functioned indistinguishably from the whole protein in suppressing the plating defect of the Δ*SooxyR* strain. In contrast, the remaining three, *So*OxyR^Δ283−^, *So*OxyR^Δ286−^, and *So*OxyR^Δ289−^, did not show any significant improvement (Fig. S6A). Interestingly, there were variations in *katB* expression in cells with these OxyR truncations. While the activating effect of *So*OxyR^Δ295−^ was the same as the intact protein, *So*OxyR^Δ292−^ showed activity of approximately 80% (Fig. 6B). Although *So*OxyR^Δ283−^, *So*OxyR^Δ286−^, and *So*OxyR^Δ289−^ were unable to fully activate expression of the *katB* gene, they exhibited H_2_O_2_-responding ability, which increased with the length of the mutated proteins. In attempts to narrow down the sequence region crucial to activity of *So*OxyR, we tested *So*OxyR^Δ290−^ and found that it was able to correct the plating defect while retaining 65% of activating capacity (Fig. 6B; Fig. S6A). These results suggest that residues from 283 to 289 are crucial for activating the function of OxyRs, while residues after 291 are dispensable. Consistent with this finding, the crystal structure of *So*OxyR RD shows that residues 284 to 290 from α10 are implicated in a dimer-dimer interaction that is critical for *So*OxyR polymerization (Fig. 5A and E).

10.1128/mbio.03497-21.6 FIG S6 Fragmentation effect of the RD domains of *So*OxyR and *Ec*OxyR for functional exchangeability. (A) Droplet assays for viability and growth assessment. (B) Impacts of the DBD domains of OxyRs on expression of *katB* by using integrative *lacZ* reporters. Cells at the mid-exponential phase were used for all assays unless otherwise noted. Cells directly taken were untreated (Un) or incubated with 0.2 mM H_2_O_2_ for 2 min and treated (T). Asterisks indicate statistically significant difference of the values compared (*n *= 4; *, *P < *0.05; **, *P < *0.01; ***, *P < *0.001). Experiments were performed at least three times, with representative results being presented. Download FIG S6, PDF file, 0.2 MB. Copyright © 2022 Sun et al. 2022 Sun et al. https://creativecommons.org/licenses/by/4.0/ This content is distributed under the terms of the Creative Commons Attribution 4.0 International license.

To verify this, we first used OxyR chimeras covering residues from 286 to 289 (Fig. 1; Table S1). Residues from 286 to 289 in *So*OxyR have the sequence of ^286^TFRT^289^, which is aligned to ^281^LYEQ^284^ in *Ec*OxyR. While OxyR^S286^^E^ and OxyR^S287^^E^ did not show a complementary effect on the plating defect, OxyR^S288^^E^ and OxyR^S289^^E^ conferred cells complete suppression (Fig. S6A). The *katB* promoter activity assays demonstrated that OxyR^S286^^E^ and OxyR^S287^^E^ could still function as a repressor in cells grown normally and, at the same time, had some ability to respond to exogenous H_2_O_2_ (Fig. 6B). In contrast, OxyR^S289^^E^ was the same as *So*OxyR, but OxyR^S288^^E^ appeared modestly impaired in activating *katB* expression. These data again testified the essential role of ^286^TFRT^289^, especially the first three residues, in the proper functioning of *So*OxyR, presumably by maintaining the correct conformation of α10 that allows tetramer formation.

To further verify the importance of residues in the proximity of R288, we carried out point mutational analyses of *So*OxyR. To begin with, we mutated R288 to E as in E. coli and P. aeruginosa (Fig. 4B). The resulting *So*OxyR_R288E_ (point mutation) was indistinguishable from *So*OxyR in terms of both viability and *katB* expression (Fig. 6B; Fig. S6A). Then, alanine scanning was conducted for residues from 285 to 291. None of these *So*OxyR mutants was significantly different from *So*OxyR in functionality (Fig. S6A and B), implying that single mutations in this region are tolerable. However, when the R288E mutation was combined with any of these alanine mutations, activity was affected substantially, depending on residues. Only one double mutant, *So*OxyR_R288E-T289A_, exhibited the characteristics of *So*OxyR, whereas all others had impaired activity (Fig. 6B; Fig. S6A and B). Among these, *So*OxyR_R288E-F287A_ and *So*OxyR_R288E-L290A_ were reduced to ∼30%, while the remaining three retained at least 60% of the wild-type activity. The crystal structure shows that R288 is at the tetramer interface, and the aliphatic portion of the R288 side chain stacks against the side chain of L285, forming part of a large hydrophobic patch that connects the two neighboring dimers together (Fig. 5D). Considering the important role of R288 in tetramer formation, it is possible that although a single mutation, R288E, can be tolerated, the simultaneous mutation of a neighboring hydrophobic residue, either F287A or L290A, would result in disruption of *So*OxyR tetramers.

### Helix 10 is critical for oligomerization upon DNA binding.

**FIG 7:**
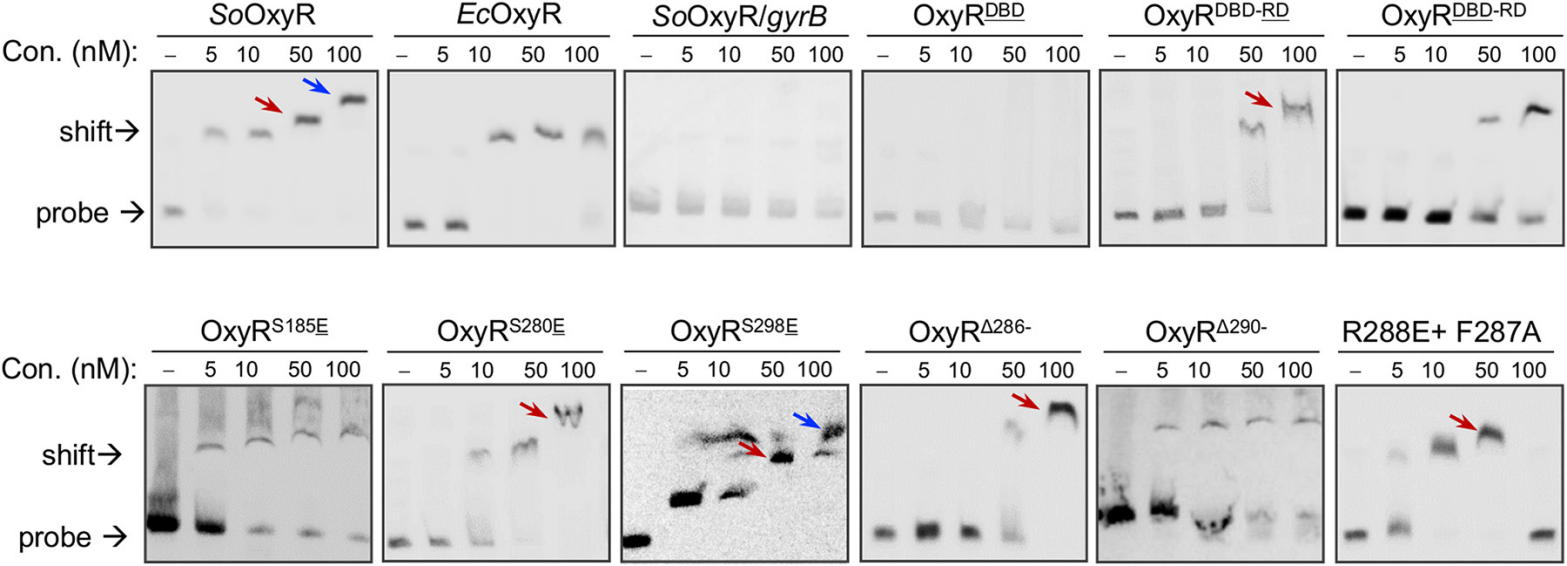
Impacts of residues within the last α-helix of *So*OxyR. *In vitro* interaction of His-tagged OxyR variants and the *katB* promoter sequence revealed by using EMSA. His_6_-tagged OxyR variants were expressed in E. coli, and proteins in soluble fractions were purified by Ni^2+^ affinity chromatography. The digoxigenin-labeled DNA probes of 188 bp that cover the OxyR-binding motif were prepared by PCR. The EMSA was performed with 10 nM probes and various amounts of proteins as indicated. The shift bands without arrow, with red arrow, and with blue arrow represent dimer, tetramer, and octamer, respectively. Experiments were performed at least three times with representative results being presented.

It is well-known that OxyR mutants impaired in DNA binding, oligomerization, or disulfide bond formation lack transcriptional activity (28). Because the last helix of OxyRs appears to be important for oligomerization based on structure analysis, we tested DNA binding of representative OxyR mutants studied above. Recombinant OxyR mutants with hexahistidine(His_6_) tag at the N terminus were expressed in E. coli and purified by Ni^2+^ affinity chromatography as before (19). Electrophoretic mobility shift assay (EMSA) results showed that both *So*OxyR and *Ec*OxyR were able to interact with the *katB* promoter, contrasting with the *gyrB* promoter used as the negative control (Fig. 7). Negative results were also obtained from the DBD domain of *So*OxyR and the target DNA fragment, indicating that they do not interact with each other specifically. Clearly, *So*OxyR differs from *Ec*OxyR in that it generates supershift bands (Fig. 7), which represent DNA oligomer (i.e., tetramer, hexamer, octamer, etc.) complexes (19). Together with the gel filtration results (Fig. S7), this observation indicates that *So*OxyR oligomerizes much more effectively than *Ec*OxyR upon binding to the *katB* promoter. Moreover, we observed a significant difference in EMSA results from OxyR^DBD-^^RD^ and OxyR^DBD^^-RD^, composed of DBD and RD from different bacteria (Fig. 7). The former, which functions as a repressor constitutively, displayed substantially impaired binding capacity, a scenario in line with the previous finding that OxyR in the activating form has a higher affinity for target DNAs than that in the repressing form (18). The latter not only was weak in binding but also impaired in oligomerization, which was supported by the data from gel filtration (Fig. S7). These observations were generally supported by results from three representative chimeric OxyR mutants, OxyR^S185^^E^, OxyR^S280^^E^, and OxyR^S298^^E^, which have no activity, repressing activity only, and full activity, respectively (Fig. 6B).

10.1128/mbio.03497-21.7 FIG S7 Gel filtration chromatogram for OxyR variants. (A) S*o*OxyR in 50 mM Tris, pH 7.4, and 50 mM NaCl and separated using a GFC-300 column. *So*OxyR gave out three well-resolved peaks (1 to 3), which corresponded to estimated masses of 290, 145, and 73 kDa, representing octamer, tetramer, and dimer, respectively. Four represents monomer. The molecular masses were estimated by the use of protein standards (*R*^2^ = 0.95). (A) Indicated OxyR variants of 10 μM in 50 mM Tris, pH 7.4, 50 mM NaCl separated using a Superdex 200 column (Pharmacia) run on an Äkta fast protein liquid chromatography (FPLC) system (Pharmacia). Download FIG S7, PDF file, 0.2 MB. Copyright © 2022 Sun et al. 2022 Sun et al. https://creativecommons.org/licenses/by/4.0/ This content is distributed under the terms of the Creative Commons Attribution 4.0 International license.

The three OxyR variants carrying mutations in the last α-helix mutants, OxyR^Δ286−^, OxyR^Δ290−^, and OxyR_R288E+F287A_, were then examined by EMSA. The former one differed from the latter two in that it could not function as an activator and retained marginal capacity for response to H_2_O_2_ (Fig. 6B). Consistently, the latter two exhibited substantially higher DNA affinity (Fig. 7). Notably, OxyR_R288E+F287A_ failed to bind to DNA probes at 100 nM (Fig. 7), an observation consistent with its reduced activating activity (Fig. 6B). More importantly, despite these differences, all of these OxyR mutants could not form octamer (Fig. 7), strongly supporting that the last helix plays an important role in oligomerization.

The crystal structure of *So*OxyR RD_C203S_ and the observation of DNA supershifts led us to a cooperative DNA-binding model by *So*OxyR (Fig. 5E). Through dimerization mediated by α10, *So*OxyR dimers can further oligomerize into tetramers, hexamers, octamers, or even larger linear complexes. In these large linear complexes, *So*OxyR dimers are related to each other by 3_2_ screw rotation symmetry as observed in the crystal structure of *So*OxyR RD_C203S_. Interestingly, two DBD domains from two adjacent *So*OxyR dimers would be brought into close proximity according to this supramolecular assembly model. *Ec*OxyR may utilize a different mechanism for the activating mode of DNA binding, thus explaining the lack of supershifts. Consistent with this model, *So*OxyR mutants with reduced or no activating activity did not produce supershifts in the DNA-binding assay.

## DISCUSSION

The purpose of this study was to unravel the mechanism underpinning the functional nonexchangeability of *Ec*OxyR and *So*OxyR, representatives of type I and II OxyRs, respectively. Given identical activating mechanisms and considerable similarity in amino acid sequences and overall structures, we anticipated that some short fragments, at least some residues, were likely responsible for the functional difference. We were surprised when it turned out not to be the case. The segment-swapping analyses indicated that the differences between *Ec*OxyR and *So*OxyR appeared to be comprehensive and profound. Based on our analyses using truncation and point mutations, we conclude that the functional irreplaceability of *So*OxyR by *Ec*OxyR cannot be easily resolved by point or short-segment swapping mutations. These results underscore the need to test more OxyRs for their ability to take the role of their counterparts in other bacteria.

*Ec*OxyR lacks repressing activity for its H_2_O_2_-responding target genes (2, 6). Given that OxyR proteins in the reduced and oxidized forms are present at the same time in the cell and repression is carried out by reduced OxyR proteins, one may imagine that *Ec*OxyR proteins in the reduced form were not mounted to levels sufficiently high to block transcription in S. oneidensis. However, the failure of *Ec*OxyR_C199S_ to repress *katB* expression eliminates this possibility. Similar scenarios about type II OxyR have been reported before in bacteria such as P. aeruginosa and Neisseria meningitidis, highlighting intrinsic differences between OxyR analogues possessing activator-only and dual-control activities (15, 29). It has been suggested that OxyR proteins may exist *in vivo* as a number of potential reaction intermediates to disulfide formation, including S‐OH, S‐NO, and S‐SG on C199 of *Ec*OxyR, offering additional options beyond an on/off redox regulation between oxidized (S‐S) and reduced (S‐H) forms (30). These intermediates may have different activities, resulting in a hierarchical response and regulation on the regulon members. As a result, repression of catalase may have something to do with a graded response to the stress. However, until now, the model of intramolecular disulfide bond formation prevails and has been widely accepted based on enormous amounts of evidence (31).

We showed here that the *So*OxyR DBD domain is essential for repression, an observation somewhat surprising because of relative higher sequence identity of the DBD domain (45% versus 35% full length) and highly similar DNA motifs for *Ec*OxyR and *So*OxyR. *Ec*OxyR and chimeric OxyR^DBD^^-RD^ (E. coli DBD) could not affect regulation, although they are able to interact with target DNAs. Despite this, the RD domain of *Ec*OxyR converts the DBD domain of *So*OxyR into a repressor. It should be noted that only a small share of the type II OxyR regulon is subjected to dual-activity regulation, indicating that type II OxyRs are able to differentiate the promoters for these regulon members from the rest of the regulon despite the high similarity of all DNA sequences to which they bind. For example, there are only 2, 1, and 1 genes under dual regulation in S. oneidensis (*katB* and *dps* encoding iron sequestering protein), P. aeruginosa (*katA* encoding major catalase), and N. meningitidis (*kat* encoding major catalase), respectively (15, 17, 29). This convinces us that activation is likely the core functioning mechanism of OxyRs, but evolution has elegantly honed these regulators for their roles in differentially mediating expression of genes containing similar DNA motifs in order to adapt to environments where they thrive.

By solving the RD domain structure of *So*OxyR, we identified significant differences between *So*OxyR and two other OxyRs, *Ec*OxyR and *Pa*OxyR, whose structures are well defined (9, 11, 18). EMSA results revealed that all OxyR mutants except those composed of the DBD domain only are able to interact with the target DNA fragment, suggesting that these proteins can properly dimerize. Clearly, this is in perfect agreement with the structural data. However, during formation of dimers of dimers, *So*OxyR displays some unique features. The overall tetramer arrangement of *So*OxyR appears to be different considerably from that of *Ec*OxyR and *Pa*OxyR. We speculate that these differences may result in distinct oligomerization status of *So*OxyR revealed in EMSA. In addition to dimer and tetramer forms, a significant share of *So*OxyR appears to be assembled into octamer (Fig. 7). As full activating activity is only observed from mutants capable of forming octamer (Fig. 7), it is reasonable to assume that this type of oligomerization is critical for *So*OxyR to act as an activator. It is worth mentioning that OxyR of P. aeruginosa (a gammaproteobacterium *per se*) is an abnormal type II OxyR because it is phylogenetically clustered with betaproteobacterial OxyRs rather than with those from *Gammaproteobacteria* (22, 29). Moreover, the last residue of the C-terminal α-helix within *Pa*OxyR is cysteine, and more importantly, this Cys residue has been suggested to be involved in peroxide sensing in P. aeruginosa (22). Thus, *Pa*OxyR is not a genuine type II OxyR defined in this study, and its deviation from *So*OxyR may not be sufficient to support the link between physiological function and structure.

Perhaps the most important finding of the structural analysis is revelation of involvement of the C-terminal α-helix in oligomerization. The loss of the entire α10 (OxyR^Δ283−^) almost completely disabled SoOxyR to respond to H_2_O_2_. However, SoOxyR without half of this α-helix (OxyR^Δ289−^) retains H_2_O_2_ response and repressing, but not activating, activity. In addition, while mutations in α10 impair both repressing and activating activities, they do not abolish the ability to respond to H_2_O_2_. Thus, α10 may not be directly associated with conformational changes induced by disulfide bond formation. EMSA results show that regardless of regulatory effects, *So*OxyR variants carrying mutations in this region lose the signature oligomerization pattern observed from the wild-type *So*OxyR, confirming the importance of α10 in assembly of tetramer and octamer. Notably, *So*OxyR variants that display activating activity are largely present in tetramer only. While this further validates the role of α10 in oligomerization, it indicates that octamer complexation is not absolutely required for activating activity.

It is also important to note that our oligomerization model (Fig. 5E) and the tetramer model proposed for *Pa*OxyR (18) and the C. glutamicum OxyR (*Cg*OxyR) (11) are not mutually exclusive. In the crystal structure of full-length *Pa*OxyR and *Cg*OxyR, two RD-interfaced homodimers further dimerize through their DBD interface to form an asymmetric tetramer. It is possible that each OxyR dimer from our linear oligomer model (Fig. 5E) is actually part of a tetramer like those observed in the *Pa*OxyR and *Cg*OxyR crystal structures. Therefore, through the C-terminal α-helix, OxyR tetramers can form bigger assemblies, such as octamers, dodecamers, etc. Indeed, octamers were observed for full-length *Cg*OxyR in solution when the protein was in the oxidized state (11). Interestingly, both full-length *Pa*OxyR and *Cg*OxyR tetramers were found to dimerize through their C-terminal α-helices in crystal.

Gene repression by type II OxyRs in bacteria has been investigated before, but the physiological relevance as to this phenomenon remains elusive (15, 29, 32). In addition to catalase, iron-sequestering protein Dps is also under dual control of OxyR in S. oneidensis (17). Given that S. oneidensis is renowned for unusually high respiratory versatility because of particular richness and abundance in iron-containing proteins, for instance, more than 40 *c*-type cytochromes, the speculation is that OxyR downregulates production of iron-containing catalase and Dps when cells are not challenged by H_2_O_2_ (19, 33, 34). In this way, the biosynthesis of iron proteins involved in metabolism and electron transport responsible for respiratory versatility gains priority so as to maintain fitness in environments (35, 36). Given that the ultimate output of transcriptional regulation is realized by interaction between regulators and their target DNAs, this complex dual control presents an extremely elaborated maneuver for tuning gene expression in response to environmental cues in different prokaryotic cells. To this end, our study represents an important step toward a better understanding of the mechanisms underlying the different functional modes of the OxyR proteins.

## MATERIALS AND METHODS

### Bacterial strains, plasmids, and culture conditions.

All bacterial strains and plasmids used in this study can be found in Table S1 in the supplemental material. Information about all of the primers is available upon request. All chemicals were obtained from Sigma-Aldrich unless otherwise noted. E. coli and S. oneidensis were grown in lysogeny broth (LB, Difco, Detroit, MI) under aerobic conditions at 37 and 30°C for genetic manipulation. When necessary, the growth medium was supplemented with chemicals at the following concentrations: 0.3 mM 2,6-diaminopimelic acid (DAP), 50 μg/mL ampicillin, 50 μg/mL kanamycin, 15 μg/mL gentamicin, 100 μg/mL streptomycin, and 2,000 U/mL catalase on plates.

Growth in liquid medium was monitored by recording values of optical density at 600 nm (OD_600_), as all strains used in this study were morphologically similar. Both LB and defined medium MS (25) were used for phenotypic assays in this study, and comparable results were obtained with respect to growth.

### Knock-in and expression of *oxyR* variants.

The mutagenesis procedure for constructing in-frame deletion (26) was used to knock in DNA sequences encoding OxyR variants after the *oxyR* promoter (P*_oxyR_*) in S. oneidensis. In brief, the target gene sequences were amplified by PCR with primers containing *attB* and the gene-specific sequence. The fragments were introduced into plasmid pHGM01 by using Gateway BP Clonase II enzyme mix (Invitrogen) according to the manufacturer’s instructions, resulting in mutagenesis vectors, which were maintained in E. coli DAP auxotroph WM3064. The vectors were subsequently transferred into the S. oneidensis Δ*oxyR* strains via conjugation. Integration of the mutagenesis constructs into the chromosome was selected by resistance to gentamicin and confirmed by PCR. Verified transconjugants were grown in LB broth in the absence of NaCl and plated on LB containing 10% sucrose. Gentamycin-sensitive and sucrose-resistant colonies were screened by PCR for deletion of the target gene. Strains carrying OxyR-variant knock-in were verified by sequencing the mutated regions.

To assess protein levels of OxyR variants, constructs for generating GFP fusion proteins were prepared. In brief, DNA fragments encoding the OxyR variants under test and *gfp* genes were PCR amplified with specifically designed primers, allowing the first-round products to be joined together by a second round of PCR as described previously (37). The final PCR products were cloned into the vectors under the control of P*_oxyR_* for knock-in as described above. Expression and localization of GFP fusions were visualized as described previously (38). For quantification of fluorescence, mid-log-phase cultures of each test strain carrying GFP fusions were collected, washed with phosphate-buffered saline containing 0.05% Tween 20, and disrupted by French pressure cell treatment. Throughout this study, the protein concentration of the resulting cell lysates was determined using a Bradford assay with bovine serum albumin (BSA) as a standard (Bio-Rad) when necessary. A volume of 100 μL cell lysates was transferred into black 96-well plates at various time intervals, and fluorescence was measured using a fluorescence microplate reader (M200 Pro Tecan) with excitation at 485 nm and detection of emission at 515 nm.

### Analysis of gene expression.

Activity of the promoter (P*_katB_*) for the major catalase *katB* gene was assessed using a single-copy integrative *lacZ* reporter system as described and used previously (39). Cells grown to the mid-exponential phase under normal or H_2_O_2_-challenging conditions (specified in the text and/or figure legends) were collected, and β-galactosidase activity was determined by monitoring color development at 420 nm using a Synergy 2 Pro200 multidetection microplate reader (Tecan), and the data were presented as Miller units.

### Droplet assays.

Droplet assays were employed to evaluate viability and growth inhibition on plates. Cells grown in LB to the mid-log phase were collected by centrifugation and adjusted to 10^9^ CFU/mL, which was set as the undiluted (dilution factor, 0). Tenfold serial dilutions were prepared with fresh LB medium. Five microliters of each dilution were dropped onto LB plates containing agents such as catalase when necessary. The plates were incubated for 24 h or longer in dark before being read. All experiments were repeated at least three times.

### Analysis of catalase.

To assess catalase levels, S. oneidensis cells grown in LB the mid-exponential phase were incubated with 0.2 mM H_2_O_2_ for 30 min and then collected by centrifugation and disrupted by French pressure cell treatment. Throughout this study, the protein concentration of the resulting cell lysates was determined using a Bradford assay with BSA as a standard (Bio-Rad). Aliquots of cell lysates containing the same amount of protein were subjected to 10% nondenaturing polyacrylamide gel electrophoresis (PAGE). Catalases were detected by using the corresponding activity-staining methods (40).

Activity of catalase was also assayed in a more quantitative approach as described previously (41). Briefly, mid-exponential-phase cells in liquid medium were collected, washed twice in 50 mM KH_2_PO_4_ buffer (pH 7.0), resuspended in the same buffer, and then disrupted by sonication. Ten microliters of cell extracts containing 40 ng/μL protein was added to 90 μL KH_2_PO_4_ and 100 μL 20 mM H_2_O_2_ in a 200-μL volume. Decomposition of H_2_O_2_ was measured at 240 nm with absorbance readings taken at 15-s time intervals for a total time of 3.5 min in a Tecan M200 Pro microplate reader. The unit of activity of each sample is expressed as μmol H_2_O_2_ decomposed per min and per mg of protein (μmol · min^−1^ · mg^−1^). Each sample was tested in quadruplicate for each strain assayed.

### Expression and purification of OxyR variants.

All OxyR variants under test were purified as His-tagged soluble proteins as described before (19). In brief, E. coli BL21(DE3) strains transformed with pET28a carrying target genes were grown in LB to the mid-log phase and then induced with 0.2 mM IPTG (isopropyl-β-d-thiogalactopyranoside) at 25°C for 6 h to produce high levels of His_6_-OxyR variants. Cell pellets were treated by French press, and His_6_-OxyR variants were purified from crude cell lysates using a nickel-ion affinity column (GE Healthcare). After removal of contaminant proteins with washing buffer containing 20 mM imidazole, the His-tagged OxyR variants were collected in elution buffer containing 100 mM imidazole. The eluted fractions were concentrated and dialyzed using a buffer containing 20 mM Tris-HCl, pH 8.0, 250 mM NaCl, 350 μL 2-mercaptoethanol (2-ME), and 1 mM NaN_3_ and further purified by gel filtration using a Superdex 200 column (Pharmacia) run on an Äkta fast protein liquid chromatography (FPLC) system (Pharmacia). The different oligomeric states of *So*OxyR were resolved with a short analytical gel filtration column (GFC 300), which allows quick injections and monitoring with great resolution. The peak fractions were collected and analyzed by SDS-PAGE. Fractions containing purified proteins were concentrated to 8 mg/mL and stored at 4°C. The identity of purified proteins was confirmed with tandem mass spectrometry (MS/MS) analysis.

### Site-directed mutagenesis.

Site-directed mutagenesis was employed to generate OxyR proteins carrying point mutations. The *oxyR* gene within the vectors used for knock-in or for expression and purification was subjected to the modification by using a QuikChange II XL site-directed mutagenesis kit (Stratagene) as described previously (42).

### Crystallization and structure determination.

Purified *So*OxyR_C203S_(point mutation) RD was crystallized at 20°C by hanging-drop vapor diffusion. Drops were made by combining 1 μL of *So*OxyR_C203S_ RD with 1 μL of mother liquor containing 4.0 M ammonium acetate and 0.1 M Bis-Tris propane, pH 7.0. Rod-shaped crystals appeared within 3 to 4 days. Crystals were harvested and flash frozen in cold nitrogen stream at −173°C using mother liquor supplemented with 20% (vol/vol) glycerol as a cryoprotectant. The diffraction data were collected from single crystals at the Life Sciences Collaborative Access Team (LS-CAT) at the Advanced Photon Source (APS). Images were collected using a 1° oscillation angle at a wavelength of 0.98 Å. The data were processed using HKL-2000 (43) (Table S2). The structure was determined by molecular replacement using the coordinates of the reduced OxyR of E. coli (PDB ID 1I69) as a search model. The structure model was built using Autobuild (PHENIX) (44) and Coot (45) and refined with phenix.refine. Merohedral twining was observed with a twining fraction of 43.5%, and therefore, the twin law (h, -h-k, -l) was applied during refinement in PHENIX. The final structure was refined to a 2.4-Å resolution (*R*_work_, 17.34%, and *R*_free_, 20.36%). There are six molecules in each asymmetric unit. All the structure figures were prepared using the program PyMOL unless otherwise specified (PyMOL Molecular Graphics System, version 2.0; Schrödinger, LLC). All structural alignment calculations were done using DALI (46). The final model of the OxyR regulatory domain S. oneidensis was deposited in the PDB with PDB ID 7L4S.

### DNA-binding analyses.

To test the interaction between OxyR and promoter regions of its target genes, electrophoretic mobility shift assays (EMSAs) were conducted as previously described (19). DNA probes covering the predicted OxyR-binding sites were obtained by PCR, during which the double-stranded product was labeled with digoxigenin-ddUTP (Roche Diagnostics). The digoxigenin-labeled DNA probes were mixed with serial dilutions of purified OxyR of various concentrations in binding buffer (4 mM Tris-HCl [pH 8.0], 40 mM NaCl, 4 mM MgCl_2_, and 4% glycerol) containing 0.75 μg of poly(dI-dC) at room temperature for 15 min. The DNA/protein mixtures were loaded on 7% native polyacrylamide gels for electrophoretic separation, and the resulting gel was visualized with the UVP image system.

### Other analyses.

Student's *t* test was performed for pairwise comparisons with statistical significance set at the 0.05 confidence level. Values were presented as means ± standard deviation.

### Data availability.

The crystallographic coordinates and associated structure factors for *S. oneidensis* OxyR are available at the Protein Data Bank (PDB) under accession code 7L4S.
