# Supplementary material for: Functional Irreplaceability of Escherichia coli and Shewanella oneidensis OxyRs Is Critically Determined by Intrinsic Differences in Oligomerization
Source: mBio. 2022 Jan 25;13(1):e03497-21. doi: 10.1128/mbio.03497-21 (PMC8787470; doi:10.1128/mbio.03497-21)
Supplement: FIG S1 [file mbio.03497-21-sf001.pdf]

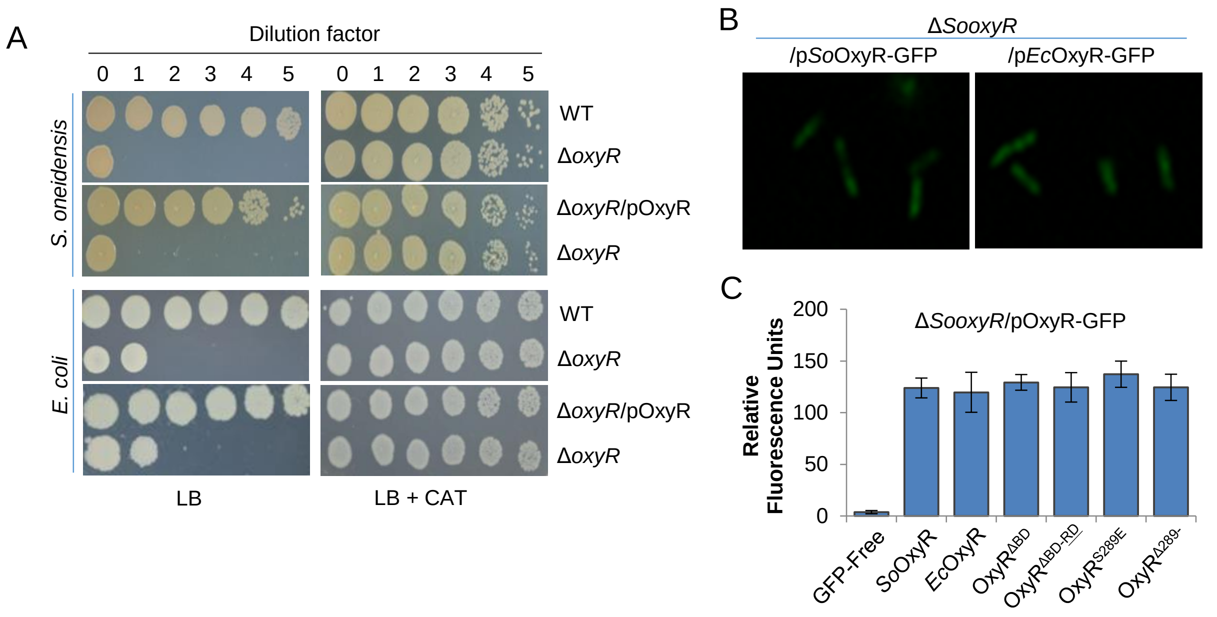

FIGURE S1. **The DBD of OxyRs alone is not functional.** *A,* Droplet assays for viability and growth assessment. Cultures of indicated strains prepared to contain approximately 10^9 cfu/ml were regarded as the undiluted (dilution factor, 0), which were subjected to 10-fold series dilution. Five microliters of each dilution was dropped on LB plates. Results were recorded after 24 h incubation. pOxyR represents that each strain expresses its own *oxyR* gene. *B,* Validation of expression of *EcoxyR* and *SooxyR* with GFP fusions. DNA constructs were placed under control of the *SoOxyR* promoter and integrated into the chromosome to allow expression from a single copy. Cells of the mid-exponential phase were visualized. *C,* GFP quantification analysis of GFP fused to indicated OxyR variants. Error bars show standard deviations. In A and B, experiments were performed at least three times, with representative results being presented.
