# Supplementary material for: Functional Irreplaceability of Escherichia coli and Shewanella oneidensis OxyRs Is Critically Determined by Intrinsic Differences in Oligomerization
Source: mBio. 2022 Jan 25;13(1):e03497-21. doi: 10.1128/mbio.03497-21 (PMC8787470; doi:10.1128/mbio.03497-21)
Supplement: TABLE S1 [file mbio.03497-21-st001.pdf]

Table S1. Strains, plasmids, and all OxyR variants used in this study

Strain or plasmid	Description	Reference or source
E. coli strains		
MG1655	Wild type	ATCC 700926
DH5α	Host for cloning	Lab stock
BL21	Recombinant protein expression host strain	Novagen
WM3064	Δ*dapA*, donor strain for conjugation	W. Metcalf, UIUC
HG-EcOxyR	Δ*oxyR* derived from MG1655	(1)
S. oneidensis strains		
MR-1	Wild type	ATCC 700550
HG1328	Δ*oxyR* derived from MR-1	(1)
HG-OxyR-v	Δ*oxyR* expressing one of all OxyR variants	This study
Plasmid		
pHGM01	Ap^r Gm^r Cm^r suicide vector	(2)
pHGEI01-P_{katB}	For measuring P_{katB} activity	(3)
pET-28a	Recombinant protein expression vector	Novagen
pET28a-OxyR-v	All OxyR variants within pET-28a	This study
OxyR variants		
*Ec*OxyR	*E. coli* wild-type OxyR	
*So*OxyR	*S. oneidensis* wild-type OxyR	
Truncations		
OxyR^{DBD}	*So*OxyR DBD domain only	
OxyR$^{\underline{DBD}}$	*Ec*OxyR DBD domain only	
*So*OxyR$^{\Delta XXX-}$	*So*OxyR lacking residues from XXX to the end	
Chimeras		
OxyR$^{DBD-\underline{RD}}$	OxyR consisting of *So*OxyR DBD and *Ec*OxyR RD domains only	
OxyR$^{\underline{DBD}-RD}$	OxyR consisting of *Ec*OxyR DBD and *So*OxyR RD domains only	
OxyR$^{SXXX\underline{E}}$	OxyR consisting of *So*OxyR sequence before residue XXX and *Ec*OxyR sequence after residue XXX	
Point mutants		
*Ec*OxyR$_{C197S}$	Residue 197 Cys replaced by Ser within *Ec*OxyR	
*So*OxyR$_{C203S}$	Residue 203 Cys replaced by Ser within *So*OxyR	
*So*OxyR$_{L285A}$	Residue 285 Leu replaced by Ala within *So*OxyR	
*So*OxyR$_{T286A}$	Residue 286 Thr replaced by Ala within *So*OxyR	
*So*OxyR$_{F287A}$	Residue 287 Phe replaced by Ala within *So*OxyR	
*So*OxyR$_{R288A}$	Residue 288 Arg replaced by Ala within *So*OxyR	
*So*OxyR$_{T289A}$	Residue 289 Thr replaced by Ala within *So*OxyR	
*So*OxyR$_{L290A}$	Residue 290 Leu replaced by Ala within *So*OxyR	
*So*OxyR$_{G291A}$	Residue 291 Gly replaced by Ala within *So*OxyR	
*So*OxyR$_{R288E}$	Residue 288 Arg replaced by Glu within *So*OxyR	
*So*OxyR$_{R288E-L285A}$	Point mutations for two residues	
*So*OxyR$_{R288E-T286A}$	Point mutations for two residues	
*So*OxyR$_{R288E-F287A}$	Point mutations for two residues	
*So*OxyR$_{R288E-T289A}$	Point mutations for two residues	
*So*OxyR$_{R288E-L290A}$	Point mutations for two residues	
*So*OxyR$_{R288E-G291A}$	Point mutations for two residues	

References:

1. Jiang Y, Dong Y, Luo Q, Li N, Wu G, Gao, H. 2014. Protection from oxidative stress relies mainly on derepression of OxyR-dependent KatB and Dps in *Shewanella oneidensis*. J Bacteriol **196**: 445-458.
2. Jin M, Jiang Y, Sun L, Yin J, Fu H, Wu G, Gao H. 2013. Unique organizational and functional features of the cytochrome *c* maturation system in *Shewanella oneidensis*. PLoS One **8**: e75610.
3. Wan F, Kong L, Gao H. 2018. Defining the binding determinants of *Shewanella oneidensis* OxyR: Implications for the link between the contracted OxyR regulon and adaptation. J Biol Chem **293**: 4085-4096.
