# Supplementary material for: Functional Irreplaceability of Escherichia coli and Shewanella oneidensis OxyRs Is Critically Determined by Intrinsic Differences in Oligomerization
Source: mBio. 2022 Jan 25;13(1):e03497-21. doi: 10.1128/mbio.03497-21 (PMC8787470; doi:10.1128/mbio.03497-21)
Supplement: TABLE S2 [file mbio.03497-21-st002.pdf]

Table S2 Crystallographic data-collection and refinement statistics

	Reduced *So*OxyR^{C203S} RD
**Data collection** [a]	
Wavelength (Å)	0.98
Space group	$P3_2$
Unit cell (Å)	a = 84.00 Å, b = 84.00 Å, c = 185.14 Å
Resolution(Å)	2.4
Unique reflections	57,110
Completeness (%)	97.9 (97.9)
Redundancy	4.1 (3.9)
Average $I/\sigma(I)$	7.7 (2.3)
R_{merge}	10.4% (50.9%)
**Refinement**	
R-factor	R_{work} = 17.5% / R_{free} = 22.0% for 5% total reflections
No. of atoms (protein/water)	9621
Rms bond length deviation	0.0054 Å
Rms bond angle deviation	0.89 °
Mean B-factor	29.0 Å^2
Ramachandran plot:	
Most favored regions (%)	95.8%
Allowed regions (%)	4.2%
Disallowed regions(%)	0%

[a] Numbers in parenthesis are for the highest resolution shell from 2.44 - 2.40 Å resolution.
