# Supplementary material for: Functional Irreplaceability of Escherichia coli and Shewanella oneidensis OxyRs Is Critically Determined by Intrinsic Differences in Oligomerization
Source: mBio. 2022 Jan 25;13(1):e03497-21. doi: 10.1128/mbio.03497-21 (PMC8787470; doi:10.1128/mbio.03497-21)
Supplement: FIG S2 [file mbio.03497-21-sf002.pdf]

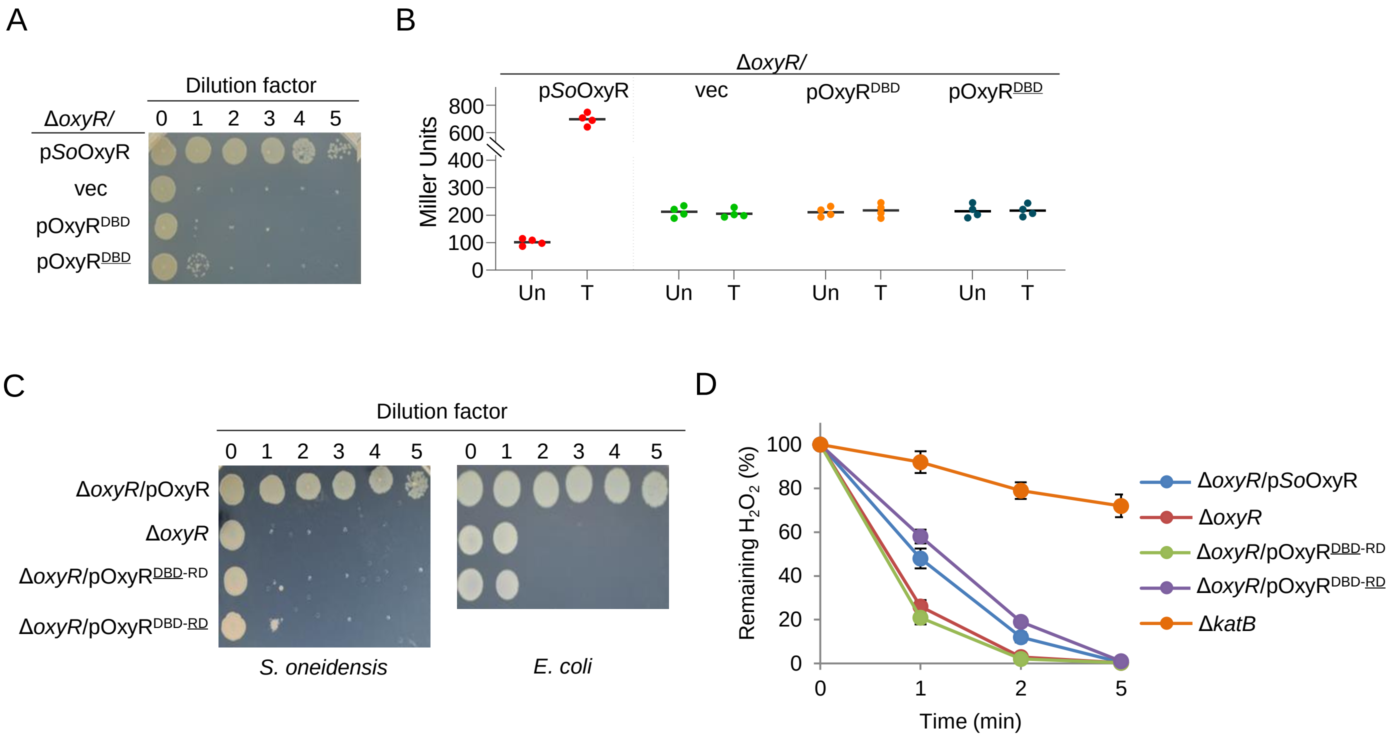

FIGURE S2. **The DBD domain of OxyRs**. The DBD domain of OxyRs has no activity in *S. oneidensis*, supported by A: Droplet assays for viability and growth assessment. $OxyR^{DBD}$ and $OxyR^{\underline{DBD}}$ represent the DBD domains of *So*OxyR and *Ec*OxyR respectively (refer to the text for details), and B: Impacts of the DBD domains of OxyRs on expression of *katB* by using integrative *lacZ*-reporters. Cells at the mid-exponential phase were used for all assays unless otherwise noted. Cells directly taken, Un-treated (Un); Incubated with 0.2 mM H_2O_2 for 2 min, treated (T). DBD of *So*OxyR is essential for repressing activity in *S. oneidensis*, supported by C: Droplet assays for viability and growth assessment, and D: H_2O_2 degradation assay. Experiments were performed at least three times, with the average $\pm$ error bars representing standard deviation or representative results being presented.
