# Supplementary material for: Functional Irreplaceability of Escherichia coli and Shewanella oneidensis OxyRs Is Critically Determined by Intrinsic Differences in Oligomerization
Source: mBio. 2022 Jan 25;13(1):e03497-21. doi: 10.1128/mbio.03497-21 (PMC8787470; doi:10.1128/mbio.03497-21)
Supplement: FIG S3 [file mbio.03497-21-sf003.pdf]

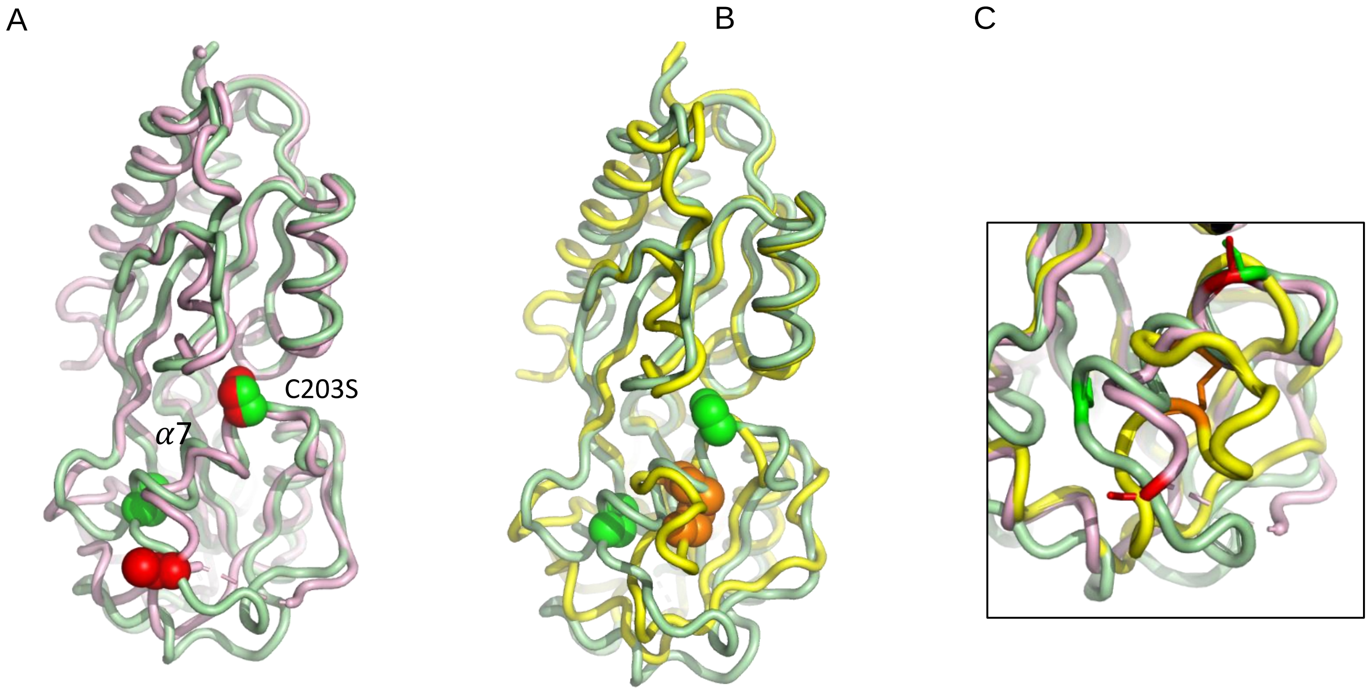

FIGURE S3. **Structural comparison of the *S. oneidensis* OxyR and *E. coli* OxyR RD.** *A*, The reduced forms of *S. oneidensis* OxyR^{C203S} RD (light green) and *E. coli* OxyR^{C199S} RD (PDB 1i69, light pink) superimposed. The redox-active cysteine residues C203S/C212 in *So*OxyR^{C203S} RD are shown in space filling model and colored in green. The redox-active cysteine residues C199S/C208 in *Ec*OxyR^{C199S} RD is also shown in space filling model but colored in red. *B*, Pairwise structure comparison of *So*OxyR^{C203S} RD (light green) and the oxidized form of *Ec*OxyR RD (PDB 1i6a, yellow). The disulfide linked C199-C208 in oxidized *Ec*OxyR RD is highlighted by orange. *C*, Comparison of the redox active centers of 'reduced' *So*OxyR^{C203S} (light green), reduced form of *Ec*OxyR^{C199S} (light pink) and oxidized form of *E. coli* OxyR (yellow). Redox-active cysteine residues C203S/C212 and C199/C208 in *So*OxyR^{C203S} and *Ec*OxyR RD, respectively, are shown in sticks representation and colored the same as in A and B.
