# Supplementary material for: Functional Irreplaceability of Escherichia coli and Shewanella oneidensis OxyRs Is Critically Determined by Intrinsic Differences in Oligomerization
Source: mBio. 2022 Jan 25;13(1):e03497-21. doi: 10.1128/mbio.03497-21 (PMC8787470; doi:10.1128/mbio.03497-21)
Supplement: FIG S4 [file mbio.03497-21-sf004.pdf]

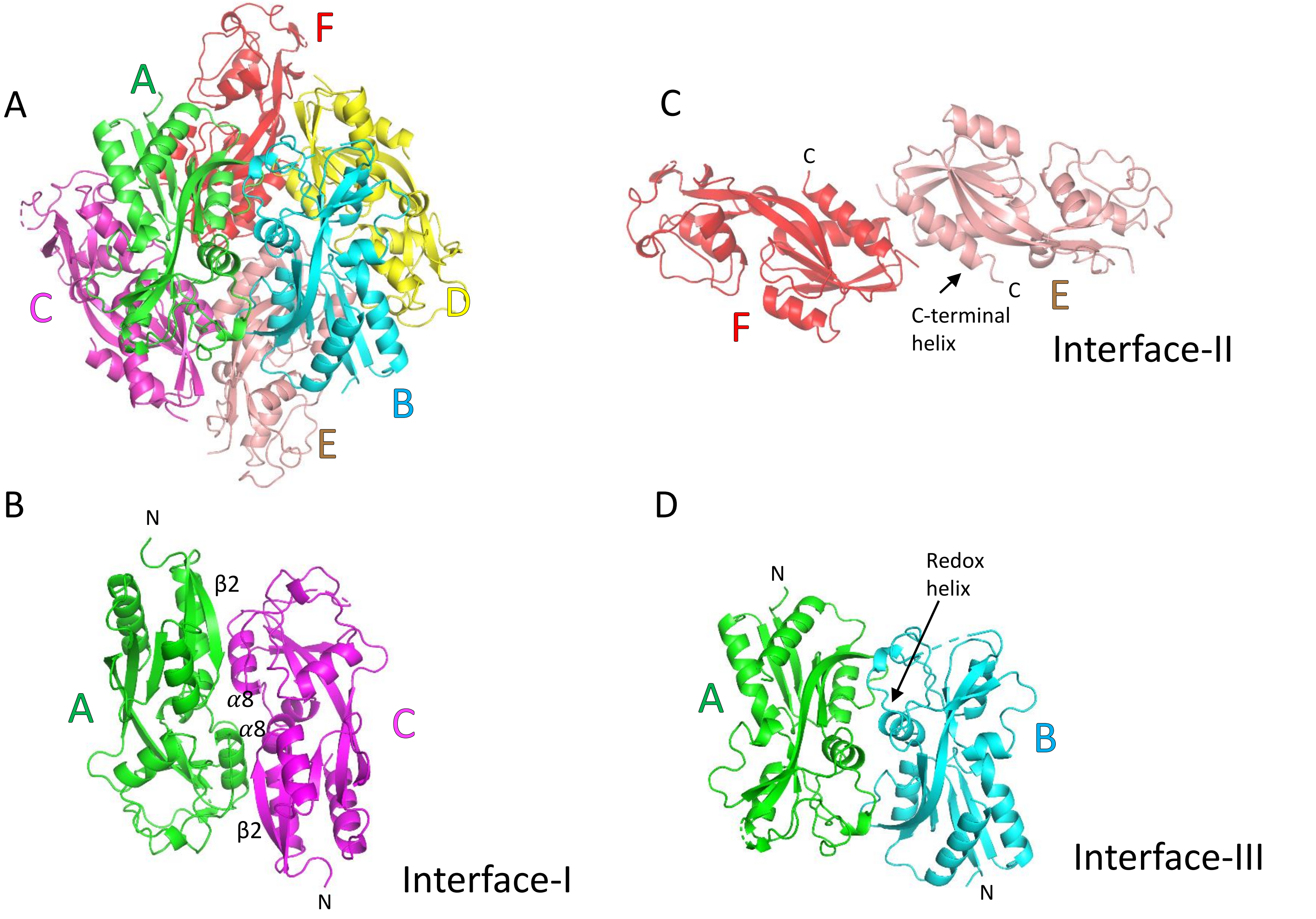

FIGURE S4. **Analysis of intermolecular interfaces**. *A,* six molecules in one crystallographic asymmetric unit. The six molecules are colored differently and labeled from A to E. *B*, Dimer by interface-I. *C*, Dimer by interface-II. *D*, Dimer by interface-III. The color scheme is consistent from *A to D*.
