# Supplementary material for: Functional Irreplaceability of Escherichia coli and Shewanella oneidensis OxyRs Is Critically Determined by Intrinsic Differences in Oligomerization
Source: mBio. 2022 Jan 25;13(1):e03497-21. doi: 10.1128/mbio.03497-21 (PMC8787470; doi:10.1128/mbio.03497-21)
Supplement: TABLE S3 [file mbio.03497-21-st003.pdf]

Table S3. Molecular interface list calculated by PDBePISA (4)

Interface	Subunit #1	Subunit #2	Symmetry operator on #2	Interface area (Å)2	Interpretation
1	D	B	x,y,z	1306.2	RD dimer (type-I)
2	C	A	x,y,z	1257.9	
3	E	F	-y+3,x-y+1,z-1/3	1239.9	
Average:				1268.0	
4	F	E	x,y,z	563.3	Dimer-dimer interface mediated by the last helix (type-II)
5	B	D	-y+2,x-y,z-1/3	560.0	
6	C	A	-y+2,x-y+1,z-1/3	558.7	
Average:				560.7	
7	B	A	x,y,z	479.2	Dimer-dimer interface involving the redox helix (type III)
8	E	C	x,y,z	464.9	Asymmetric
9	F	A	x,y,z	227.8	Asymmetric
10	E	B	x,y,z	214.6	Asymmetric
11	F	D	x,y,z	186.1	Asymmetric
12	B	C	x,y-1,z	144.3	Asymmetric
13	A	D	x-1,y,z	2.4	Asymmetric
14	A	F	-y+2,x-y,z-1/3	140.4	Asymmetric
15	B	F	-y+2,x-y,z-1/3	103.7	Asymmetric
16	E	D	x,y,z	57.7	Asymmetric
17	E	B	-y+2,x-y+1,z-1/3	25.1	Asymmetric
18	F	C	x,y,z	18.7	Asymmetric
19	E	A	-y+2,x-y+1,z-1/3	1.0	Asymmetric

References:

4. Krissinel E. Henrick K. 2007. Protein interfaces, surfaces and assemblies' service PISA at the European Bioinformatics Institute. *'Inference of macromolecular assemblies from crystalline state.'*. *J Mol Biol* **372**: 774—797.
