# Supplementary material for: Functional Irreplaceability of Escherichia coli and Shewanella oneidensis OxyRs Is Critically Determined by Intrinsic Differences in Oligomerization
Source: mBio. 2022 Jan 25;13(1):e03497-21. doi: 10.1128/mbio.03497-21 (PMC8787470; doi:10.1128/mbio.03497-21)
Supplement: FIG S5 [file mbio.03497-21-sf005.pdf]

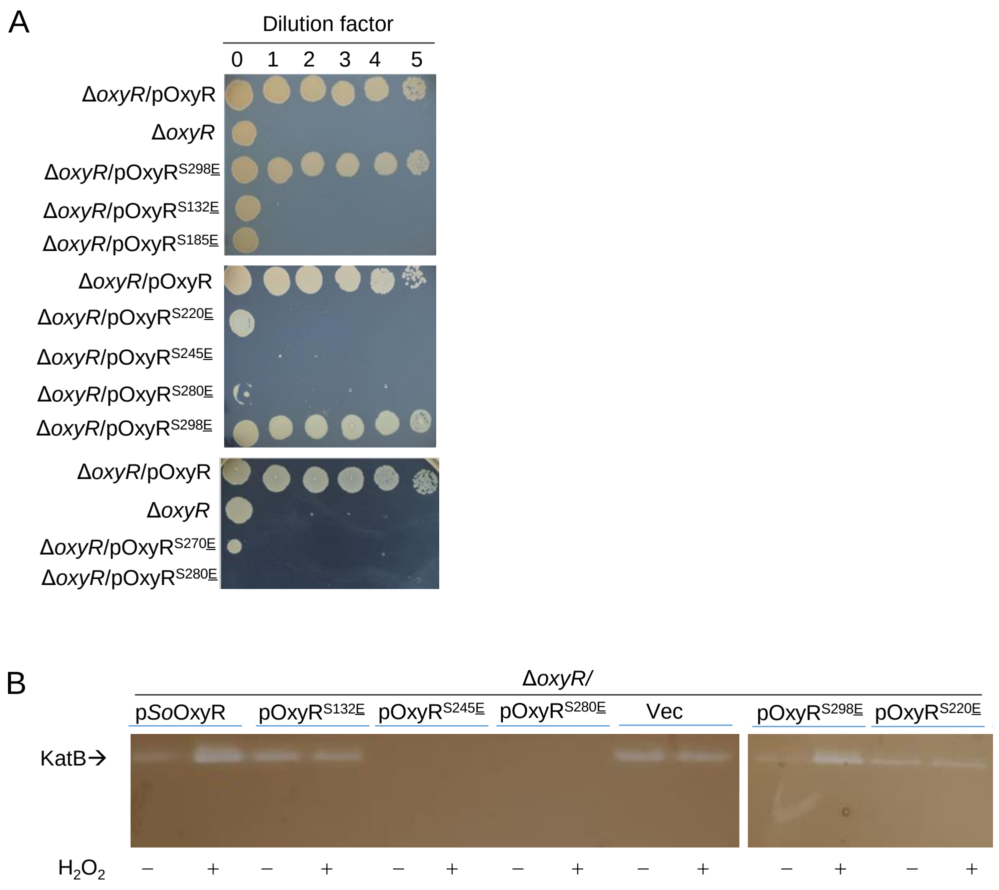

FIGURE S5. **Fragmentation effect of the RD domains of *So*OxyR and *Ec*OxyR for functional exchangeability**. *A,* Droplet assays for viability and growth assessment. *B*, Catalase detected by staining and activity assay. Cells were either directly used or incubated with 0.2 mM H_2O_2 for 30 min. Experiments were performed at least three times, with representative results being presented.
