# Supplementary material for: Functional Irreplaceability of Escherichia coli and Shewanella oneidensis OxyRs Is Critically Determined by Intrinsic Differences in Oligomerization
Source: mBio. 2022 Jan 25;13(1):e03497-21. doi: 10.1128/mbio.03497-21 (PMC8787470; doi:10.1128/mbio.03497-21)
Supplement: FIG S6 [file mbio.03497-21-sf006.pdf]

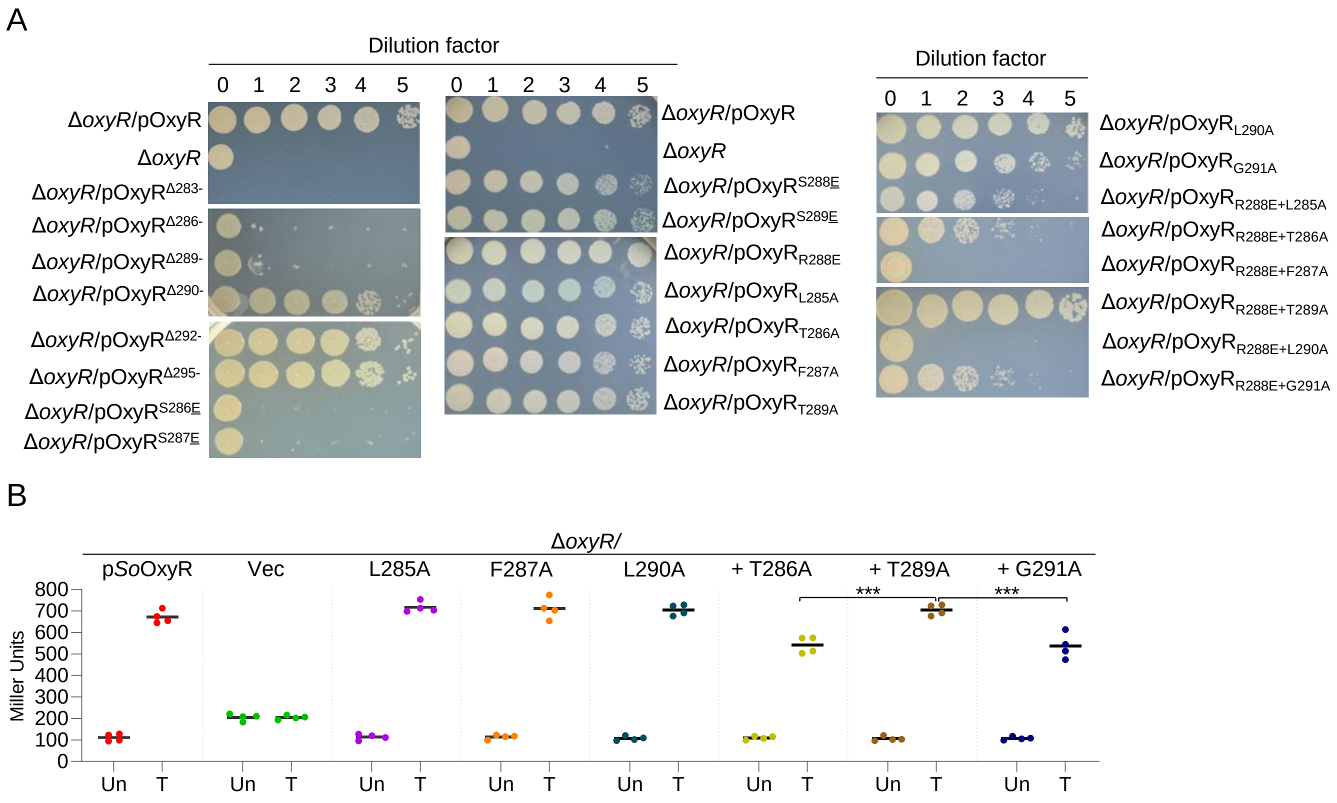


FIGURE S6. **Fragmentation effect of the RD domains of *So*OxyR and *Ec*OxyR for functional exchangeability**. *A*, Droplet assays for viability and growth assessment. *B*, Impacts of the DBD domains of OxyRs on expression of *katB* by using integrative *lacZ*-reporters. Cells at the mid-exponential phase were used for all assays unless otherwise noted. Cells directly taken, Un-treated (Un); Incubated with 0.2 mM $H_2O_2$ for 2 min, treated (T). Asterisks indicate statistically significant difference of the values compared ($n = 4$, *, $P < 0.05$; **, $P < 0.01$; ***, $P < 0.001$). Experiments were performed at least three times, with representative results being presented.
