# Supplementary material for: Functional Irreplaceability of Escherichia coli and Shewanella oneidensis OxyRs Is Critically Determined by Intrinsic Differences in Oligomerization
Source: mBio. 2022 Jan 25;13(1):e03497-21. doi: 10.1128/mbio.03497-21 (PMC8787470; doi:10.1128/mbio.03497-21)
Supplement: FIG S7 [file mbio.03497-21-sf007.pdf]

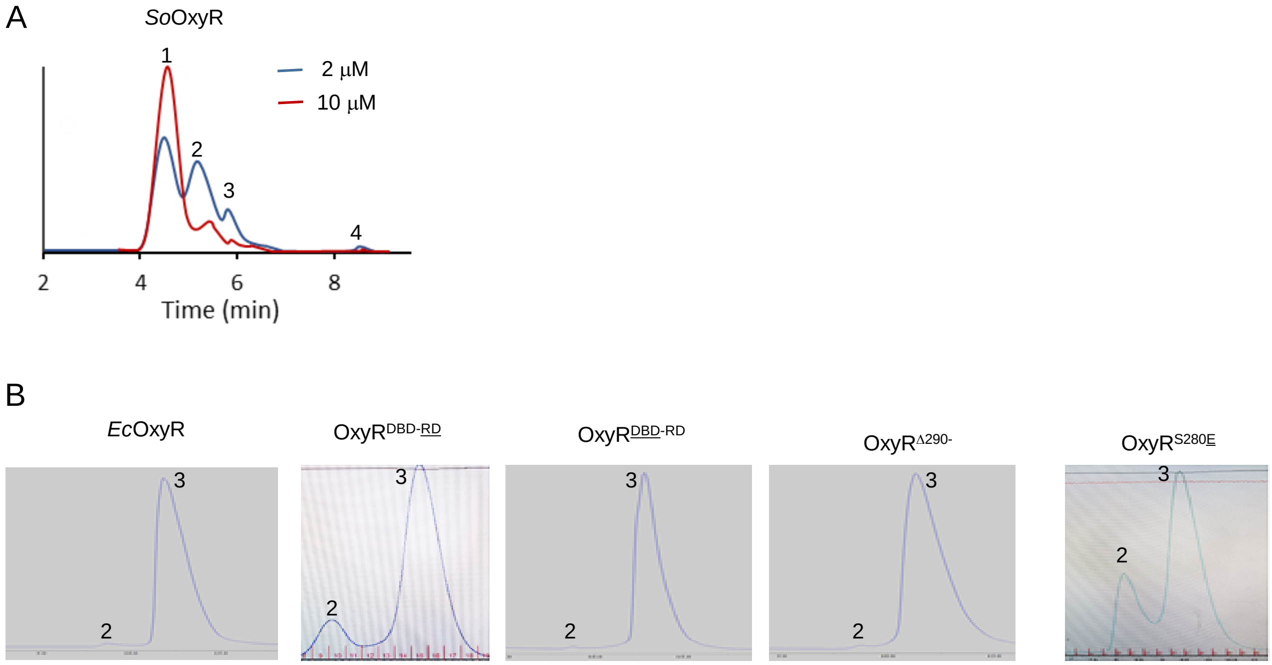

FIGURE S7. **Gel filtration chromatogram for OxyR variants.** *A*, *So*OxyR in 50 mM Tris pH 7.4, 50 mM NaCl separated using GFC-300 column. *So*OxyR gave out three well-resolved peaks (1, 2, 3), which corresponded to estimated masses of 290, 145, and 73 kDa, representing octamer, tetramer, and dimer, respectively. 4 represents monomer. The molecular masses were estimated by the use of protein standards (R^2 = 0.95). *A*, Indicated OxyR variants of 10 μM in 50 mM Tris pH 7.4, 50 mM NaCl separated using a Superdex 200 column (Pharmacia) run on an Äkta FPLC system (Pharmacia).
